# Puf3 contributes to changes in mRNA solubility, translation elongation dynamics at rare arginine codons and loss of protein homeostasis in cells lacking Not4

**DOI:** 10.1261/rna.080953.126

**Published:** 2026-09

**Authors:** Léna Audebert, George E. Allen, Siyu Chen, Olesya O. Panasenko, Susanne Huch, Christine Polte, Zoya Ignatova, Vicent Pelechano, Martine A. Collart

**Affiliations:** 1 Department of Microbiology and Molecular Medicine, Faculty of Medicine and Institute of Genetics and Genomics Geneva, University of Geneva, 1211 Geneva 4, Switzerland; 2 The BioCode: RNA to Proteins Core Facility, Faculty of Medicine, University of Geneva, 1211 Geneva 4, Switzerland; 3 SciLifeLab, Department of Microbiology, Tumor and Cell Biology, Karolinska Institutet, 17165 Solna, Sweden; 4 Biochemistry and Molecular Biology, University of Hamburg, 20146 Hamburg, Germany

**Keywords:** Not4, Puf3, ribosome dwelling, rare arginine codons, protein homeostasis, mRNA solubility

## Abstract

The Not proteins of the Ccr4-Not complex regulate translation elongation dynamics, essential for proper folding and assembly of new proteins. In yeast, ribosomes with nonoptimal codons in the A-site are enriched within the pool of ribosomes bound by Not4 and Not5. Such ribosomes accumulate in cells lacking Not4 or Not5 that show defects in co-translational assembly and aggregation of new proteins. Recently, we observed that depletion of Not1 and Not4 inversely regulate changes in mRNA solubility, correlating with inverse codon-specific changes in A-site ribosome dwelling occupancies (RDOs). Here we describe that mRNAs less soluble upon Not4 depletion are enriched for targets of the RNA-binding protein Puf3. We determine that Puf3 contributes to differential changes of A-site RDOs upon Not1 and Not4 depletion, in particular at rare arginine codons, and it contributes to changes in mRNA solubility in *not4*Δ. Moreover, deletion of Puf3 suppresses temperature sensitivity and protein aggregation in the *not4*Δ strain, while overexpression of Puf3 is toxic. Interestingly, the Puf3 interactome is altered in *not4*Δ. Taken together, our results associate alterations in Puf3 to *not4* mutant phenotypes, in particular to changes in translation elongation dynamics and protein aggregation.

## INTRODUCTION

The Ccr4-Not complex is a global regulator of mRNA metabolism that is conserved in all eukaryotic cells (Collart 2016). It is built upon a central scaffold protein Not1, onto which other subunits dock organized in functional modules (Maillet et al. 2000; Basquin et al. 2012; Bhaskar et al. 2013, 2015; Boland et al. 2013; Sgromo et al. 2017). It is mostly described to repress gene expression when recruited to its target mRNAs, either by leading to mRNA deadenylation (Bartlam and Yamamoto 2010; Stowell et al. 2016; Raisch and Valkov 2022; Pavanello et al. 2023) or by interacting with factors that block translation initiation (Okada et al. 2015; Kuzuoglu-Ozturk et al. 2016; Wilczynska et al. 2019; Rasch et al. 2020; Guenole et al. 2022). However, accumulating evidence in the last 15 years indicate that it also plays a critical role for translation elongation dynamics (Panasenko and Collart 2011, 2012; Halter et al. 2014; Gupta et al. 2016; Kassem et al. 2017; Allen et al. 2021, 2023; Chen et al. 2023) and facilitates folding and co-translational assembly of proteins. The Ccr4-Not complex can be recruited to its target mRNAs by RNA-binding proteins (Chekulaeva et al. 2011; Vindry et al. 2012; Fabian et al. 2013; Pekovic et al. 2023), and also by interacting with translating ribosomes (Buschauer et al. 2020). Not5, in association with Not4, binds to the E site of posttranslocation ribosomes when A sites are empty, a situation more likely to occur when a nonoptimal codon is present in the A site. Thereby, it has been proposed that the Ccr4-Not complex monitors the mRNA turnover according to codon optimality (Buschauer et al. 2020). We have proposed instead a model whereby binding of the Not proteins to the translating ribosome modulates translation elongation dynamics, to facilitate co-translational events (Allen et al. 2021). Notably, the two models are not mutually exclusive. The Not proteins form cytosolic condensates, named assemblysomes, and in human cells, CNOT1 condensates promote colocalization of mRNAs whose nascent peptides interact co-translationally (Panasenko et al. 2019). We thus proposed that condensation of Not-associated translating ribosomes regulates translation elongation dynamics (Allen et al. 2021). Following up on this model, we determined that mRNA solubility in budding yeast varies with distinct translation elongation dynamics for soluble and insoluble mRNAs (Allen et al. 2021, 2023). Solubility refers to the relative presence of a given mRNA in a total cell lysate prepared under conditions of salt and detergent typical of polysome and ribosome profiling experiments used to characterize translation elongation dynamics, compared to its relative presence in the total RNA pool that can be extracted from a cell pellet by acid phenol. We did not characterize what makes an mRNA less soluble, but one can expect that mRNAs functionally associated with membranes or in condensates associated or not with membranes, but also mRNAs in aggregates, will tend to be less soluble. In any event, we noted that ribosomes dwelling at nonoptimal A site codons reside more on insoluble mRNAs than on soluble ones (Allen et al. 2023), indicating a link between mRNA solubility and translation elongation dynamics, a link that we could further connect to the function of the Ccr4-Not complex. Indeed, we determined that depletion of two different subunits of the Ccr4-Not complex, the scaffold protein Not1 or the E3 ligase subunit Not4, inversely modified mRNA solubilities correlating with opposing effects on translation elongation dynamics (Allen et al. 2023). Consistent with their role in translation elongation dynamics, the Not proteins have been described as essential for preventing the aggregation of newly synthesized proteins (Halter et al. 2014). In this context, it is reasonable to speculate that the solubility of mRNA and the process of translation elongation are connected, and that this connection is crucial in preventing the aggregation of newly synthesized proteins.

Substrates of Not4-dependent ubiquitination have variable stability (for review, see Collart 2013), and even when stable steady state levels of a given ubiquitinated substrate is detected in cells, as is the case for the ribosomal protein Rps7a, cycles of ubiquitination and deubiquitination can be critical for function, in this case for ensuring proper rounds of translation (Takehara et al. 2021). Thereby, the immediate depletion of Not4 that abolishes new ubiquitination events but does not impact immediately the levels of stable ubiquitinated substrates of Not4, has different translation elongation phenotypes compared to the total deletion of Not4 in which none of the substrates are ubiquitinated, and moreover, adaptations can occur (Allen et al. 2023). In this work, we zoomed into mRNAs less soluble upon Not4 depletion but more soluble upon Not1 depletion, and determined that they were enriched for targets of the Puf3 pumilio family RNA-binding protein (RBP) (Olivas and Parker 2000). Puf3 is a key regulator of mitochondrial mRNAs and acts at the posttranscriptional level through binding to a consensus sequence (UGUA_AUA) (Webster et al. 2019) in 3′UTRs (Kershaw et al. 2015). It has many mitochondrial targets (Gerber et al. 2004; Rowe et al. 2014; Kershaw et al. 2015; Lapointe et al. 2018; Liu et al. 2021), but many transcripts regulated by Puf3 are not linked to mitochondria (Kershaw et al. 2015; Webster et al. 2019). Moreover, Puf3 binds and regulates transcripts with noncanonical binding sites (Lapointe et al. 2018), though motif quality is the major determinant of Puf3-dependent mRNA stability for Puf3 targets in vivo (Webster et al. 2019). Puf3 promotes binding of the Ccr4-Not complex to mRNA isoforms with Puf3 binding sites (Gupta et al. 2016) to accelerate their deadenylation (Lee et al. 2010; Webster et al. 2019), in particular during fermentation. Nonfermentable sugar conditions inhibit Puf3's ability to accelerate mRNA deadenylation within minutes, via phosphorylation of Caf1, a Ccr4-Not subunit, by Yak1 that abolishes the interaction between Puf3 and the Ccr4-Not complex (Bulmash et al. 2023). During respiratory conditions, Puf3 stabilizes mRNAs that encode mitochondrial proteins (Bulmash et al. 2023) and contributes to their translation to support the need for increased mitochondrial biogenesis. It has been proposed that differential phosphorylation of Puf3's large N-terminal intrinsically disordered region, and Puf3 condensation, are components of a regulatory system that maintains Puf3 as a repressor under fermentation conditions but transforms it into an activator for preexisting mRNAs upon switch to respiratory conditions (Miller et al. 2014; Lee and Tu 2015).

Our current results uncover a codon-specific role of Puf3 in translation elongation dynamics during fermentation and a surprising dominant negative function of Puf3 in *not4*Δ, where it contributes to deregulating translation elongation dynamics and solubility of mRNAs, leading to temperature sensitivity and protein aggregation. They suggest that Not4's role in protein homeostasis relates at least in part to its role in ensuring appropriate interactome and functional control of Puf3.

## RESULTS

### Puf3 contributes to the solubility of mRNAs in cells lacking Not4

**FIGURE 1.**
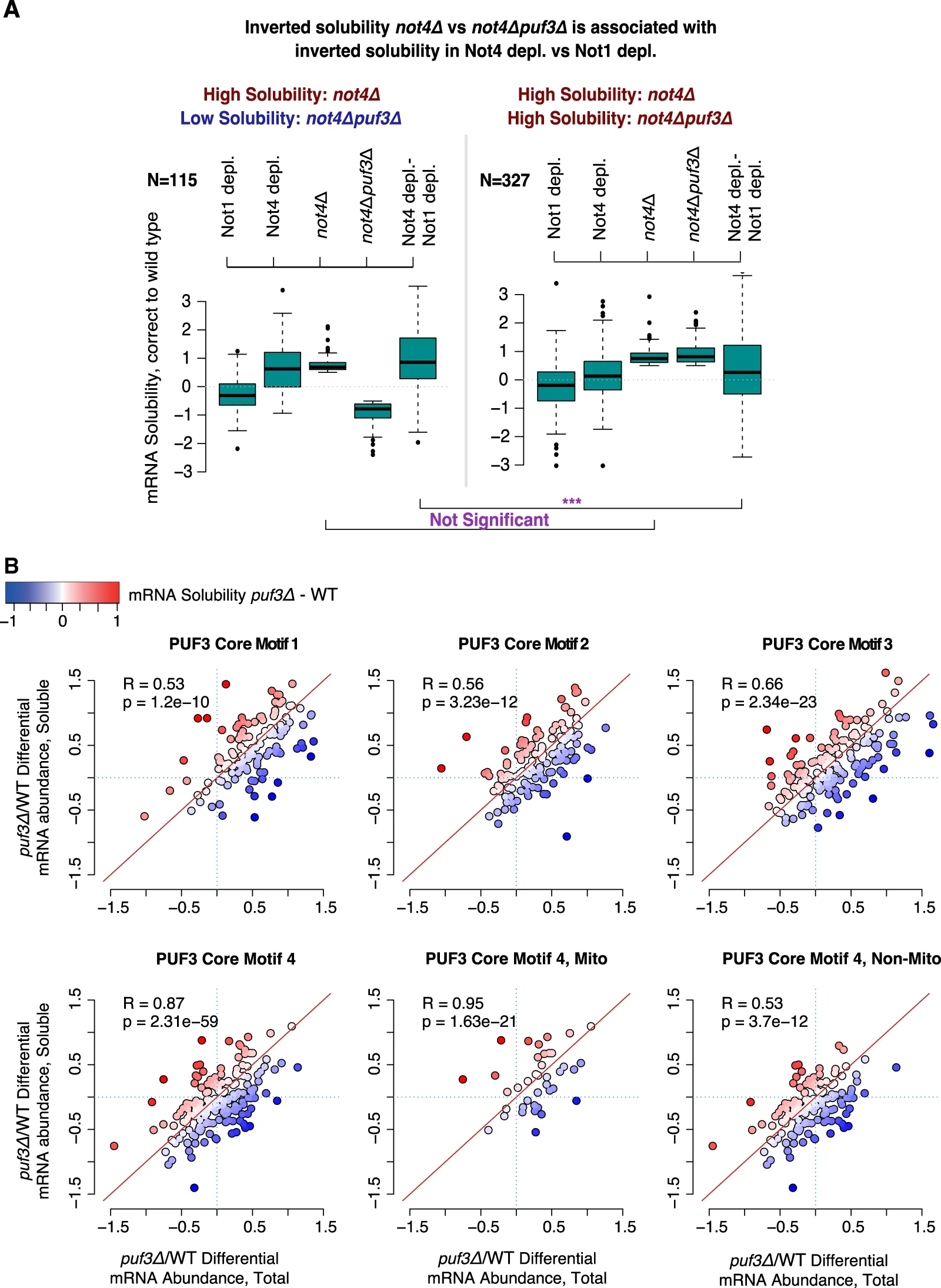
Inverted mRNA solubility in *not4*Δ versus *not4*Δ*puf3*Δ is associated with inverted solubility upon Not4 depletion vs upon Not1 depletion. (*A*) The 115 mRNAs that show an increase in solubility in *not4*Δ compared to wild type (a log_2_ fold change higher than 0.5), but a decrease in solubility in the *not4*Δ*puf3*Δ (log_2_ fold change lower than −0.5) are represented in the *left* panel. The 327 mRNAs that show an increase in solubility in *not4*Δ compared to wild type and also in *not4*Δ*puf3*Δ compared to wild type (log_2_ fold change higher than 0.5) are represented in the *right* panel. The change in solubility of each pool of mRNAs upon Not1 and Not4 depletion is represented in the first and second columns of each panel, and the difference in change of solubility of each pool of mRNAs upon Not1 and Not4 depletion is shown in the *far-right* column of each panel. A Welch's *t*-test was used to perform the statistical analysis. This shows that the solubility of the 115 mRNAs of the *left* panel is significantly inversely regulated upon Not1 and Not4 depletion compared to the 327 mRNAs whose solubility is not differentially impacted upon Not1 and Not4 depletion. (*B*) mRNA solubility differences in *puf3*Δ versus wild type (log_2_ ratio of mRNA abundance in *puf3*Δ versus wild type in the lysate [*y*-axis] versus in the total RNA pool [*x*-axis]) is indicated by the color of the dots. Each panel represents Puf3 target subsets with binding motifs more or less close to the Puf3 consensus binding site (according to Webster et al. 2019). There is no overall shift in abundance of mRNAs in the soluble versus total RNA pools, in the mutant versus the wild type, regardless of the quality of the Puf3 consensus motif.

When analyzing our previously published data (Allen et al. 2023), we found that Puf3 targets were enriched within the pool of mRNAs whose solubility was increased upon Not1 depletion but decreased upon Not4 depletion (Supplemental Fig. S1A; Allen et al. 2023). Intrigued by this observation, we looked at the impact of Puf3 on solubility of mRNAs in cells lacking Not4 altogether (see strain list in Supplemental Table S1). As before (Allen et al. 2023), we performed RNA-seq on the total RNA extracted from cell pellets by acid phenol and on soluble mRNA pools from cell lysates. We evaluated solubility, defined as the log_2_ fold change in expression in the soluble RNA-seq data set relative to total RNA-seq expression. We created boxplots of solubilities comparing transcripts where *not4*Δ and *not4*Δ*puf3*Δ have opposite effects, to those where their effects are similar. We observed that, among mRNAs which gain solubility in *not4*Δ (Fig. 1A), some reverse this change in *not4*Δ*puf3*Δ (Fig. 1A, left panel) and some do not (Fig. 1A, right panel). Symmetrically, it was the same among mRNAs which lose solubility in *not4*Δ (Supplemental Fig. S1B): some reverse this change in *not4*Δ*puf3*Δ (Supplemental Fig. S1B, right panel) and some do not (Supplemental Fig. S1B, left panel). Notably, the inverse changes in solubilities upon Not4 and Not1 depletions (Fig. 1A; Supplemental Fig. S1B, first two columns of left and right panels) was significantly higher for mRNAs whose changes in solubility in *not4*Δ was reversed in *not4*Δ*puf3*Δ compared to those that were not (Fig. 1A; Supplemental Fig. S1B, compare last columns of right and left panels).

We also assessed the effect of Puf3 absence on expression and solubility of mRNAs in a wild type Not4 context, focusing in particular on Puf3 targets according to the conservation of the Puf3 consensus motif (Fig. 1B; Webster et al. 2019). As previously reported (Webster et al. 2019), Puf3 target mRNAs were more likely to be upregulated in the absence of Puf3 if the mRNAs carry a conserved consensus motif (compare category 1 to nonmitochondrial category 4 target mRNAs in Fig. 1B), but we observed no overall shift in mRNA solubility in cells lacking Puf3, regardless of the quality of the Puf3 consensus motif.

Taken together, these results indicate that Puf3 does not specifically contribute to the solubility of its own target mRNAs, but it can reverse changes of solubility due to the absence of Not4 for a small number of mRNAs, in particular those inversely regulated by Not1 and Not4.

### Puf3 contributes to protein aggregation and temperature sensitivity in cells lacking Not4

As mentioned above, previous studies have indicated that Not4 is an important player in protein homeostasis and that new proteins aggregate in the absence of Not4 (Panasenko and Collart 2012; Halter et al. 2014; Preissler et al. 2015). We also reported that Not proteins form condensates, and we proposed that condensation of mRNAs by the Not proteins, potentially regulating mRNA solubility as we define it (see above), could be an active mechanism during translation elongation important for protein homeostasis (Allen et al. 2021). For instance, this condensation of mRNAs during translation elongation might regulate time or accessibility of the nascent chain to protein partners or chaperones for proper protein folding and/or complex assembly (Allen et al. 2021).

**FIGURE 2.**
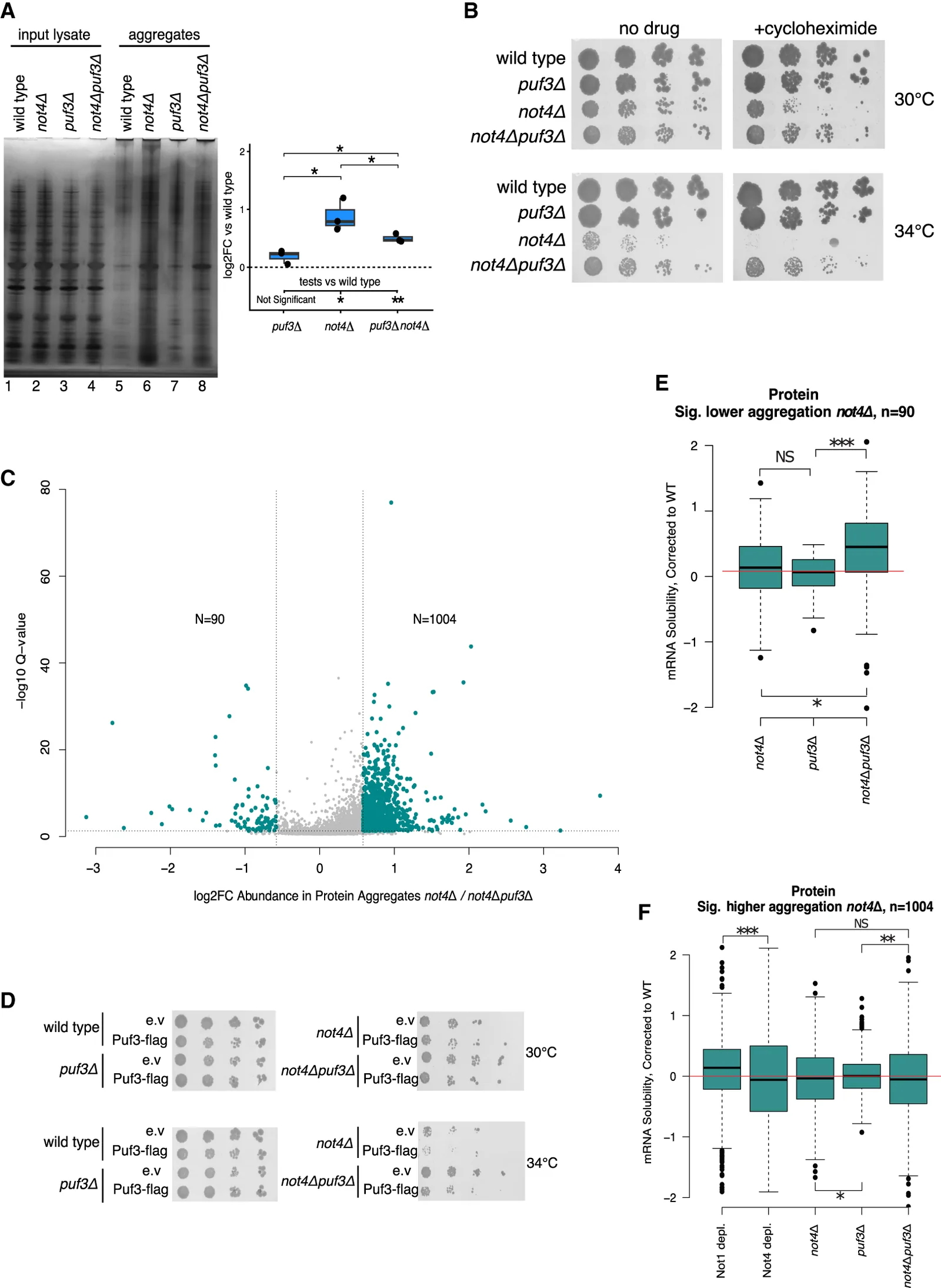
Puf3 contributes to protein aggregation and temperature sensitivity of *not4*Δ cells. (*A*) Protein aggregates (lanes *5*–*8*, *left* panel) were isolated from total cellular lysates (input lysate, lanes *1*–*4*, *left* panel) prepared from wild type, *not4*Δ, *puf3*Δ and *not4*Δ*puf3*Δ cells and analyzed by SDS-PAGE and silver staining. Quantification (*n* = 4) of the total protein in aggregates (silver stain signal in lanes *5*–*8*, *left* panel) was performed by ImageJ and is shown in the *right* panel. Fold changes relative to wild type were log_2_-transformed to stabilize variance and approximate normality. Boxplots display the distribution of log_2_ fold changes, with individual replicates overlaid, pairwise significance indicated above the boxes (*P < 0.05, **P < 0.01, ***P < 0.001), and comparisons against wild type shown below. (*B*) The indicated cells were serially diluted and grown for 4 days in the presence or absence of cycloheximide (6 μg/mL) on rich medium at 30°C or 34°C, as indicated. (*C*) Protein aggregation is reduced upon deletion of Puf3 in *not4*Δ, as demonstrated by the differential quantitative abundance of the identified proteins in the two strains, represented in a volcano plot (log_2_FC denotes the log_2_ fold change). (*D*) The indicated cells were transformed with a plasmid expressing Puf3-flag or an empty vector (e.v.) as a control. They were serially diluted and left to grow on medium selective for the plasmid for 7 days at the indicated temperatures. (*E*) Box plot comparing the 90 genes encoding proteins that aggregate significantly less in *not4*Δ compared to the double *not4*Δ*puf3*Δ mutant (“Sig. lower aggregation”) with regard to changes in solubility of the 90 encoding mRNAs (*y*-axis) in *not4*Δ, *puf3*Δ or *not4*Δ*puf3*Δ compared to the wild type. Significance of the solubility changes between columns is indicated by * (NS, not significant). (*F*) Box plot as in *E* but for the 1004 genes whose encoded proteins aggregate significantly more in *not4*Δ compared to the double *not4*Δ*puf3*Δ mutant (“Sig. higher aggregation”). Changes of mRNA solubility upon Not1 or Not4 depletion for the same sets of mRNAs are indicated in the first two columns.

We thus questioned whether the deletion of Puf3 that alters mRNA solubility in *not4*Δ (see above Fig. 1A), also had an impact on protein aggregation in this mutant. We prepared cell lysates from which we isolated aggregated proteins, for wild type cells, single *puf3*Δ and *not4*Δ mutants and the double *not4*Δ*puf3*Δ mutant. We analyzed the profile of proteins in the total lysate and in the aggregates by SDS-PAGE and silver staining. The profile of total proteins in cell lysates was similar in all four strains (Fig. 2A, left panel, lanes 1–4). However, comparing the same amount of total protein lysate, there were few proteins in aggregates from wild type cells (Fig. 2A, left panel, lane 5) compared to much higher protein levels in aggregates from cells lacking Not4 (Fig. 2A, left panel, lane 6). In cells lacking Puf3, the level of protein aggregation was comparable to that in wild type cells (Fig. 2A, left panel, compare lane 7 to lane 5). The deletion of Puf3 in *not4*Δ reduced protein aggregation, however, to levels that remained higher than in wild type cells (Fig. 2A, compare lane 8 to lanes 6 and 5 and see right panel for quantification).

To determine whether the differences in protein aggregation levels was functionally relevant, we compared the growth of the different strains at a higher temperature, when the pressure for protein folding is increased. Mirroring the differences in protein aggregation, we noted that *not4*Δ was temperature sensitive, corroborating earlier observations (Halter et al. 2014), while this sensitivity was partially suppressed at 34°C by the additional deletion of Puf3 (Fig. 2B, left panels). The deletion of Puf3 also suppressed the reduced growth that *not4*Δ cells display in the presence of the translation elongation inhibitor cycloheximide (Fig. 2B, right panels).

We next identified and compared the proteins in aggregates in *not4*Δ and in *not4*Δ*puf3*Δ by quantitative mass spectrometry (Supplemental Fig. S2A; Supplemental Table S2). Only a few proteins aggregated more in the double mutant compared to the single mutant (Fig. 2C, 90 proteins with a *Q* < 0.05 and a log_2_ fold change <−0.5 on the left of the volcano plot), whereas instead a great number of proteins aggregated less in the double mutant compared to the single mutant (Fig. 2C, 1004 proteins with a *Q* < 0.05 and a log_2_ fold change >0.5 on the right of the volcano plot). Hence, these results confirm that Puf3 contributes to protein aggregation in cells lacking Not4.

Since the deletion of Puf3 has a suppression effect in *not4*Δ, we tested the consequence of overexpressing Puf3 in *not4*Δ at 30°C and 34°C (Fig. 2D). Overexpression of Puf3-flag on a centromeric plasmid with its own promoter and 3′UTR had no detectable effect on the cell growth of wild type cells or of cells lacking Puf3, but it was toxic in *not4*Δ and to a greater degree in *not4*Δ*puf3*Δ at 34°C Thus, Puf3 has a dominant negative effect in *not4*Δ.

### Changes in protein aggregation and mRNA solubility do not generally correlate when Puf3 is deleted in *not4*Δ

As we propose that appropriate location and solubility of certain mRNAs could be important for translational events and the proper folding of proteins, we next addressed whether differences in mRNA solubility between the *not4*Δ single mutant and the *not4*Δ*puf3*Δ double mutant correlated with changes in protein aggregation.

We focused on the 90 proteins that aggregated more in the double mutant compared to the single mutant (Supplemental Table S2). Overall, their mRNAs tended to be more soluble in the double mutant cells (Fig. 2E), and they were highly enriched for mRNAs that encode interactors of the Hsp90 chaperone (Truman et al. 2015) and targets of Puf3 (Supplemental Fig. S2B; Supplemental Table S3; Webster et al. 2019). This observation indicates that Not4 and Puf3 lower the solubility of certain mRNAs, thereby helping to keep their encoded proteins from aggregating. It is notable that while some interactors of Hsp90 are enriched within this category, it is a specific subset, as overall Hsp90 interactors tend to be less aggregated in the double mutant compared to the single mutant (Supplemental Fig. S2C) and mRNAs encoding Hsp90 interactors are also overall less soluble in *not4*Δ than in the double mutant (Supplemental Fig. S2D). This is true for mRNAs encoding Hsp90 itself (Hsc82 and Hsp82) that in addition are less soluble in *not4*Δ than in the wild type (Allen et al. 2023). Similarly, for the enriched Puf3 target mRNAs whose encoded proteins aggregate more in the double mutant than in the single mutant, they were more soluble in the double than in the single mutant, but they represent only a subset of Puf3 target mRNAs that show no overall trend (Supplemental Fig. S2E).

We then looked at the mRNA set encoding proteins that aggregated less in the double mutant cells (Supplemental Table S2). They did not show a particular common trend regarding changes in mRNA solubility. Interestingly, however, these were mRNAs showing slight but significant opposite changes in solubility upon Not1 and Not4 depletion, and they were mRNAs which are less soluble in *puf3*Δ*not4*Δ than in *puf3*Δ (Fig. 2F).

### Puf3 deletion reduces accumulation of ribosomes at the stop codon in *not4*Δ

Since the mRNAs encoding proteins that aggregated less in the double mutant compared to *not4*Δ did not display any specific trend in mRNA solubility, and given that proper translation elongation is critical for the correct folding of certain proteins (for review, see Sherman and Qian 2013), we performed Ribo-seq analysis comparing wild type cells upon Not1 or Not4 depletion, in the presence and absence of Puf3. We confirmed the efficient depletion of Not4 in the experiment set up (Supplemental Fig. S3A).

**FIGURE 3.**
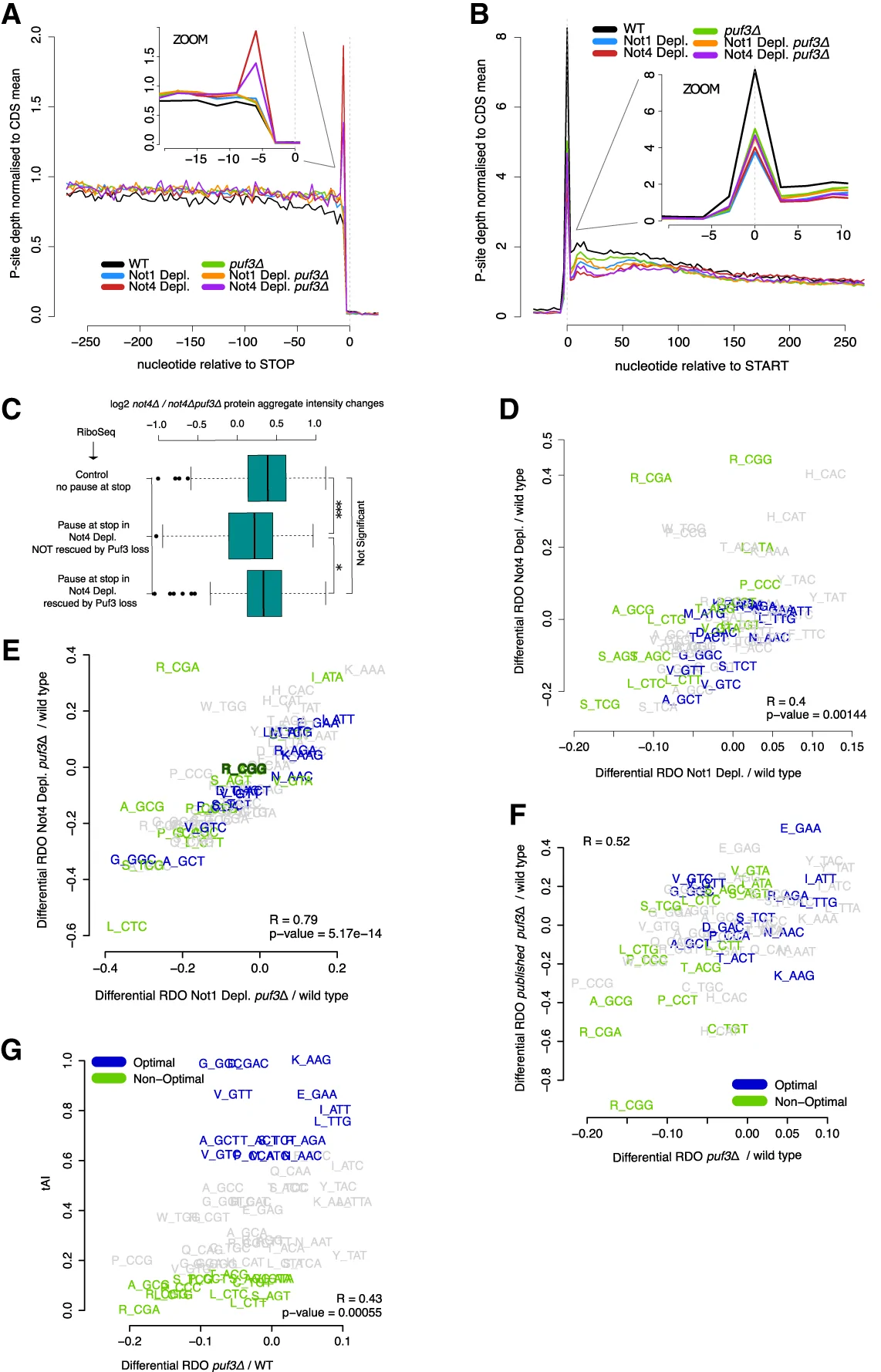
Difference in changes of codon-specific A-site ribosome dwelling occupancies upon Not1 and Not4 depletion are in large part due to Puf3. (*A*,*B*). Metagene analysis in wild type and upon Not1 or Not4 depletion with or without Puf3, extracted from Ribo-seq analysis, comparing ribosome dwelling at the stop codon (*A*) and at the start codon (*B*) is depicted. For changes in ribosome dwelling at the stop codon, paired *t*-tests comparing normalized P-site depth, one codon upstream of Stop shows: Not4 depletion versus WT: *P* < 5 × 10^−324^, Not4 depletion *puf3*Δ versus WT: *P* = 8.4 × 10^−93^, Not4 depletion versus Not4 depletion *puf3*Δ: *P* = 2.5 × 10^−76^. (*C*) Box plot of log_2_ fold changes in protein aggregation in *not4*Δ versus *not4*Δ*puf3*Δ for proteins derived from mRNAs that show no increased ribosome pausing at stop (*top* line) and those that do upon Not4 depletion. The latter group is further split into mRNAs where the pause is rescued (*bottom* line) or not (*middle* line) in the double mutant. A two-sided Welch's *t*-test was used to perform all pairwise comparisons. This reveals an inverse relationship between suppression of pausing at stop upon Not4 depletion by *puf3*Δ and suppression of protein aggregation in *not4*Δ by *puf3*Δ. (*D*–*G*) Scatterplot analyses comparing codon-specific changes in A-site ribosome dwelling occupancies, upon Not1 and Not4 depletion (*D*); upon Not1 and Not4 depletion in cells lacking Puf3 (*E*); for *puf3*Δ cells growing in glucose (*x*-axis) compared to *puf3*Δ cells transferred to respiratory growth (Wang et al. 2019) (*y*-axis) (*F*); and finally for *puf3*Δ cells growing in glucose (*x*-axis) compared to the tRNA adaptation index (tAI, *y*-axis) (Pechmann and Frydman 2013) with a weak but noticeable correlation (*R* = 0.43) (*G*). In the figure, depletion is referred to by Depl. The 15 most optimal codons in budding yeast are indicated in blue and the 15 most nonoptimal codons are depicted in green, according to Pechmann and Frydman (2013). (Gray) All other codons.

Metagene analyses for the different strains revealed that ribosomes accumulated at the stop codon specifically upon depletion of Not4, as we observed previously when sequencing decay intermediates using the 5′P sequencing technique (Allen et al. 2023) that allows identification of the last translating ribosome (Pelechano et al. 2015). Interestingly, this increase was partially suppressed upon deletion of Puf3 (Fig. 3A). At the start codon, ribosomes accumulated less in all of the mutant strains compared to the wild type (Fig. 3B).

We compared the mRNAs according to the change in accumulation of ribosomes at the stop codon upon Not4 depletion in the presence and absence of Puf3, regarding differences in protein aggregation of encoded proteins and mRNA solubility in *not4*Δ versus *not4*Δ*puf3*Δ. We observed that there was no correlation between changes in mRNA solubility and protein aggregation in *not4*Δ versus *not4*Δ*puf3*Δ overall, nor for the subset of mRNAs whose increase of ribosomes at the stop codon upon Not4 depletion was suppressed by *puf3*Δ (Supplemental Fig. S3B). However, we made the interesting observation that for the mRNAs where the increased ribosome dwelling at the stop codon upon Not4 depletion was not rescued by Puf3 deletion (i.e., persisted), the encoded proteins aggregated less in *not4*Δ than in the double mutant (Fig. 3C, middle line) compared to the mRNAs where the increased ribosome dwelling at the stop codon was rescued (Fig. 3C, bottom line). This observation suggests a connection between increased ribosome pausing at stop upon Not4 depletion and the risk for protein aggregation in the absence of Not4.

Taken together, these results support the existence of a Not4-Puf3 axis that coordinates ribosome pausing at the stop codon and proteostasis for specific mRNAs.

### Puf3 affects translation elongation dynamics related to codon optimality

As we know that for certain mRNAs, appropriate ribosome pausing is important for co-translational events (Chen et al. 2023), we next evaluated codon-specific changes in A-site ribosome dwelling occupancies (RDOs) in the different strains. In the presence of Puf3, changes in A-site RDOs showed only minimal correlation upon Not1 and Not4 depletion, with notably significant differences in dwelling at the two rare arginine codons, CGA and CGG (Fig. 3D, *R* = 0.4). Indeed, we detected higher RDOs at CGG or CGA codons in the A site upon Not4 depletion, which remained either unaffected or decreased upon Not1 depletion. In Puf3 absence, the overall correlation between changes in codon-specific A-site RDOs upon Not1 and Not4 depletion markedly increased (Fig. 3E, *R* = 0.79). These findings indicate that Puf3 affects differences in A-site RDOs upon Not1 and Not4 depletion. Importantly, Puf3 contributed significantly to differences in changes of A-site RDOs upon Not1 and Not4 depletion for mRNAs, whether or not they were targets of Puf3 (Supplemental Fig. S3C). Of note, the A-site RDOs at rare arginine codons upon Not4 depletion were more markedly increased for mRNAs that are not targets of Puf3 (Supplemental Fig. S3C, upper left panel) than for Puf3 targets (Supplemental Fig. S3C, lower left panel), whereas in Puf3 targets the A-site RDOs decreased more markedly at rare arginine codons upon Not1 depletion and Puf3 deletion (Supplemental Fig. S3C, lower right panel) compared to non-Puf3 targets (Supplemental Fig. S3C, upper right panel).

Our findings suggest that, under conditions of impaired Not protein function, Puf3 modulates translation elongation dynamics in yeast cells growing fermentatively, conditions in which Puf3 was previously believed to primarily regulate its target mRNAs through degradation or translational repression (Bulmash et al. 2023 and references therein). To investigate whether this regulatory effect persists when the Not proteins’ functions are intact, we analyzed codon-specific A-site RDO changes between wild type and *puf3*Δ cells cultured in glucose. The deletion of Puf3 had a codon-specific effect on A-site RDO changes in cells growing fermentatively. They correlated (*R* = 0.52) with codon-specific A-site RDO changes between wild type and *puf3*Δ cells grown under respiratory conditions, as calculated from published ribosome profiling data (Fig. 3F; Wang et al. 2019). Interestingly, reduced A-site RDOs at rare arginine codons was the most significant change observed in cells lacking Puf3 compared to a wild type. In addition, there was a weak but noticeable correlation (*R* = 0.43) between changes in A-site RDOs and codon optimality (Fig. 3G). For the first time, we demonstrate that Puf3 modulates translation elongation dynamics in a codon optimality-dependent manner. This regulation occurs both independently of, and in concert with, the Not4 protein and does not specifically affect the Puf3 target mRNAs.

### Dwelling at rare arginine codons decreased in *not4*Δ*puf3*Δ relative to *not4*Δ but only for mRNAs encoding proteins that aggregate less in *not4*Δ*puf3*Δ relative to *not4*Δ

The findings above show that changes in A-site RDOs at rare arginine codons is the most noticeable change upon Not4 depletion (increased) and in the absence of Puf3 (decreased). To determine how codon-specific changes in A-site RDOs are related to changes in protein aggregation between *not4*Δ and *not4*Δ*puf3*Δ, we compared two equal sets of 300 mRNAs. The first group are the 300 mRNAs encoding proteins that aggregate in *not4*Δ but show the least change in aggregation of encoded proteins in the double mutant. The second group are the 300 mRNAs encoding proteins that aggregate in *not4*Δ but exhibit the greatest reduction in aggregation of encoded proteins in the double mutant. For these two categories, we computed the A-site RDO changes in the double mutant (depletion of Not4 and *puf3*Δ) relative to the single mutant (depletion of Not4). For the mRNAs whose encoded proteins show the greatest reduction in protein aggregation in *not4*Δ*puf3*Δ relative to *not4*Δ, the A-site RDOs at cysteine TGC codons were higher in the double mutant than in the single mutant (*P* = 0.0153), whereas the A-site RDOs at the rare CGA and CGG arginine codons showed the greatest decrease (Supplemental Fig. S4A). These results indicate that ribosome dwelling at rare arginine codons fine-tune the production of soluble proteins. This regulation is disrupted by Not4 depletion but partially rescued upon additional deletion of Puf3.

**FIGURE 4.**
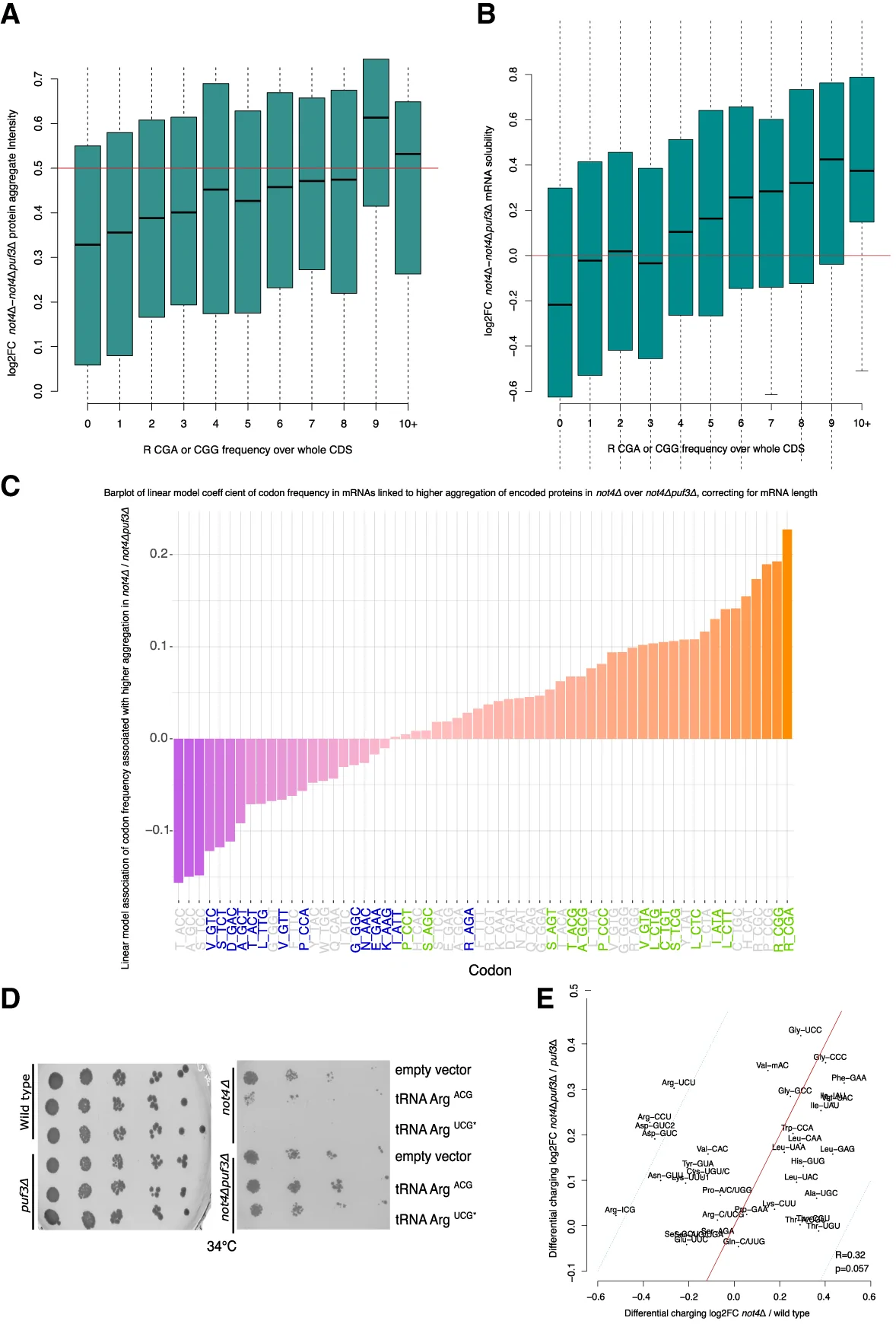
A-site RDOs at rare arginine codons upon Not4 depletion reversed by the deletion of Puf3 are associated with aggregation of encoded proteins. (*A*) mRNAs whose encoded proteins were detected in *not4*Δ aggregates were classified according to their rare arginine codon content (number of such codons in the mRNA) (CGG and CGA, *x*-axis) and changes in aggregation of encoded proteins in the single compared to the double mutant evaluated (*y*-axis). Results shown by box plot analysis indicate that the more the mRNAs contain rare arginine codons, the more the encoded proteins aggregate in *not4*Δ compared to *not4*Δ*puf3*Δ. (*B*) As in *A* except that it is changes in mRNA solubility in the double compared to the single mutant that is compared (*y*-axis). This shows that the more the mRNAs contain rare arginine codons, the more the mRNAs are soluble in *not4*Δ compared to the double mutant. (*C*) Linear model of codon frequency in mRNAs that encode proteins that aggregate more in *not4*Δ than in *not4*Δ *puf3*Δ, correcting for transcript length, indicates a trend of mRNA content in optimal versus nonoptimal codons relating to differential aggregation of encoded proteins in single and double mutant. The 15 most optimal codons in budding yeast are indicated in blue and the 15 most nonoptimal codons are depicted in green, according to Pechmann and Frydman (2013). (Gray) All other codons. (*D*) Spot assay (6 days growth on medium selective for the plasmid, at 34°C) comparing the growth of wild type, *puf3*Δ, *not4*Δ and *not4*Δ*puf3*Δ cells transformed with a centromeric plasmid bearing the tRNA having the ACG anticodon (tRNAArg^ACG^), the tRNA having an anticodon that can directly decode CGA (tRNAArg^UCG*^), or an empty vector (pRS416) as a control. (*E*) Scatterplot comparing the change in tRNA charging in *not4*Δ compared to wild type (*x*-axis) or in *not4*Δ*puf3*Δ compared to *puf3*Δ (*y*-axis) (see Supplemental Fig. S4F). This shows a differential arginine charging effect when deleting *NOT4* in a *puf3*Δ background compared to a wild type background.

We thus classified mRNAs encoding proteins that aggregate in *not4*Δ according to their rare arginine codon content and evaluated the relative aggregation of the encoded proteins in *not4*Δ and in *not4*Δ*puf3*Δ mutants (Fig. 4A). We observed more aggregation in the single mutant than in the double mutant proportional to the amount of rare arginine codons. Since one might expect that longer mRNAs may have increased chances to bear more rare arginine codons, and that mRNAs with rare arginine codons are more likely to be less expressed, we evaluated changes in A-site RDOs at rare arginine codons across broad expression levels and mRNA lengths upon Not4 depletion (Supplemental Fig. S4B) and upon Not4 depletion in the additional absence of Puf3 (Supplemental Fig. S4C) and compared them to wild type. Upon Not4 depletion, A-site RDOs increased at rare arginine codons, and relatively decreased in the double mutant, for mRNAs at all expression levels and lengths. This was suppressed by *puf3*Δ also at all expression levels and lengths (there are more blue dots, indicating less pausing, across the *x* and *y* axes in Supplemental Fig. S4C compared to Supplemental Fig. S4B). We then addressed whether the number of arginine codons had an impact on the relative solubility of mRNAs in the single *not4*Δ mutant versus the double *not4*Δ*puf3*Δ mutant (Fig. 4B). Indeed, mRNAs with more arginine codons tended to be more soluble in the single mutant compared to the double mutant relative to mRNAs with less arginine codons.

Finally, since both ribosome occupancy at the stop codon upon Not4 depletion and the rare arginine codon content of the mRNA is related to mRNA solubility and aggregation of encoded proteins in *not4*Δ, we analyzed whether mRNAs with increased ribosome occupancy at the stop codon also bear rare arginine codons. There was no correlation between the number of rare arginine codons and pausing at the stop codon. Equally, there was no significant change in ribosome 3 nt periodicity comparing wild type to Not4 depletion with or without Puf3, when comparing transcripts with a twofold increase of ribosome occupancy at stop upon Not4 depletion relative to wild type and those showing minor changes (−0.1 < log_2_FC < 0.1) (data not shown). Interestingly, the 500 top rare Arg codon-containing mRNAs encode proteins aggregating in *not4*Δ, but to a lesser extent in *not4*Δ*puf3*Δ (Supplemental Table S3). To consolidate these observations, we further investigated the role of codon content of mRNAs in the aggregation propensity of their encoded protein products in the single versus double mutant. This was done using a linear model of codon frequency in mRNAs against the relative aggregation of their encoded protein products in the single versus double mutant, correcting for transcript length. This analysis also revealed the most prominent role for rare arginine codons and additionally a bias related to codon optimality (Fig. 4C).

Taken together, these results indicate that proteins produced from mRNAs with more rare arginine codons aggregate less in *not4*Δ*puf3*Δ compared to *not4*Δ, their encoding mRNAs are overall less soluble in the double mutant relative to the single mutant, and they exhibit lower A-site RDOs at the rare arginine codons upon Not4 depletion with the additional absence of Puf3. These results indicate that Not4 and Puf3 oppositely regulate ribosome dwelling at rare arginine codons in association with differences in mRNA solubility, thereby preventing aggregation of the encoded proteins.

### Puf3 contributes to aggregation of the arginyl-tRNA synthetase in *not4*Δ

Ribosomes with nonoptimal codons in the A-site, including the nonoptimal arginine codons, are enriched within the pool of ribosomes bound by Not5, in complex with Not4 (Buschauer et al. 2020), and they accumulate in cells lacking Not4 or Not5 (Allen et al. 2021) or when Not4 is rapidly depleted according to Ribo-seq (see above). A possible explanation for this enrichment could be the reduced availability of the cognate aminoacyl-tRNAs in the mutant cells. We thus tested whether increasing the expression of tRNAs decoding rare arginine codons might improve growth of *not4*Δ. We overexpressed a tRNA^Arg^ACG that decodes the CGA codon. This tRNA is encoded by six genes in yeast and we have chosen the gene *YNCE0028C*. A34 in the ACG anticodon is modified to inosine, so that tRNA^Arg^ICG decodes CGU, CGC, and CGA, as described in Wada and Ito (2023). The yeast genome does not encode a tRNA^Arg^ with the UCG anticodon. Nevertheless, to not depend on the A34 modification, we also mutated the anticodon of tRNA^Arg^ACG and created a unique tRNA^Arg^UCG directly pairing to the CGA codon. The expression of the additional tRNA genes was toxic in *not4*Δ*,* particularly for the CGA modified tRNA, while the deletion of Puf3 suppressed the toxic effect at 34°C (Fig. 4D). There was no toxicity observed in wild type or *puf3*Δ.

In *not4*Δ we have previously observed that the amount of arginyl-tRNAs^Arg^ was generally reduced, while the levels of the uncharged tRNAs^Arg^ were unchanged (Allen et al. 2021), suggesting that charging of tRNAs^Arg^ might be impaired in *not4*Δ. We also noted that the arginine-tRNA synthetase (or ligase), encoded by *YDR341C*, catalyzing the charging of all tRNAs^Arg^, aggregated in *not4*Δ, while its aggregation in the double mutant was significantly reduced (Supplemental Fig. S4D). This observation raised the question as to whether availability of arginyl-tRNAs^Arg^ might explain the differences in A-site RDOs at rare arginine codons. Therefore, using tRNA-tailored microarrays, we measured charging levels of all tRNAs in *puf3*Δ or in *puf3*Δ*not4*Δ (Supplemental Fig. S4E,F; Supplemental Table S4) and compared them to previous measurements in wild type and *not4*Δ (Allen et al. 2021). Notably, the levels of arginyl-tRNA^Arg^ were improved or unchanged in the double *puf3*Δ*not4*Δ mutant compared to the single *puf3*Δ mutant (Fig. 4E). Hence in the background of *puf3*Δ, we do not see that *not4*Δ results in decreased arginyl-tRNA^Arg^ as we do see in the presence of Puf3. Since the level of charged tRNAs is the most potent factor affecting A-site RDOs (Pavlov et al. 2021; Davyt et al. 2023), it provides a plausible explanation for the lower RDOs observed by depleting Not4 in the absence of Puf3.

Charging of tRNAs depends also upon amino acid availability. Hence, we next focused on the *CPA1* gene—encoding a protein participating in arginine biosynthesis—whose expression is affected by arginine availability. This regulation occurs via production of a leader peptide from an upstream ORF (uORF) (Delbecq et al. 1994). Following Not4 depletion, we observed accumulation of ribosome-protected fragments in the uORF, compatible with an excess of available arginine. This however was not detectably different in the double mutant or even exaggerated in cells lacking Puf3 (Supplemental Fig. S4G, left). Consistent with a suppressant effect of uORF expression on the expression of the ORF, we see a commensurate significant loss in the CDS P-site PPKMs (P-sites per kilobase million) upon Not4 depletion, with or without the additional deletion of Puf3 (Supplemental Fig. S4G, right). Hence, availability of arginine is high and similar in single and double mutants, which does not explain the differential RDOs observed.

### Differences in the Puf3 interactome in cells with or without Not4

Our results above show that Puf3 affects translation elongation dynamics (in particular at rare arginine codons) alone or in combination with Not4. Thereby, it has a dominant negative impact in cells lacking Not4. We thus decided to purify Puf3 from both wild type and *not4*Δ cells to observe if the interactome in *not4*Δ could explain some of the toxicity mediated by Puf3 in the absence of Not4. We used cells expressing a tagged version of Puf3 (i.e., Puf3-TAP) and used the Tap-tag for affinity purification. Puf3-TAP was slightly less expressed in *not4*Δ compared to wild type (Supplemental Fig. S5A). It was very difficult to purify native Puf3 from cell lysates from *not4*Δ (Supplemental Fig. S5B). However, when we mixed wild type cells expressing untagged Puf3 with *not4*Δ cells expressing Puf3-TAP (using equivalent OD_600_ cell counts of each), recovered the mixed cells by centrifugation and then broke them together with glass beads and lysis buffer, we obtained more intact Puf3-TAP protein (Supplemental Fig. S5B).

We decided to use this mixing approach to purify Puf3-TAP and compare its interactome in wild type and *not4*Δ. For purifications, all lysates originate from the same mixed pool of wild type and *not4*Δ cells, but Puf3 is Tap-tagged either only in the wild type cells (condition 1) or in the *not4*Δ cells (condition 2). We also used as a negative control mixed wild type cells and *not4*Δ cells lacking any tagged protein (condition 3). Since the lysates in all three conditions have the same total protein content, differences in the output of the purifications are not expected to be due to interactions in the test tube, but to represent differential purification of Puf3 interactors from the strain where Puf3 was tagged. For the analyses, we thus focused on proteins that were differentially present in the purifications of Puf3-TAP from wild type and *not4*Δ and absent in the negative control.

**FIGURE 5.**
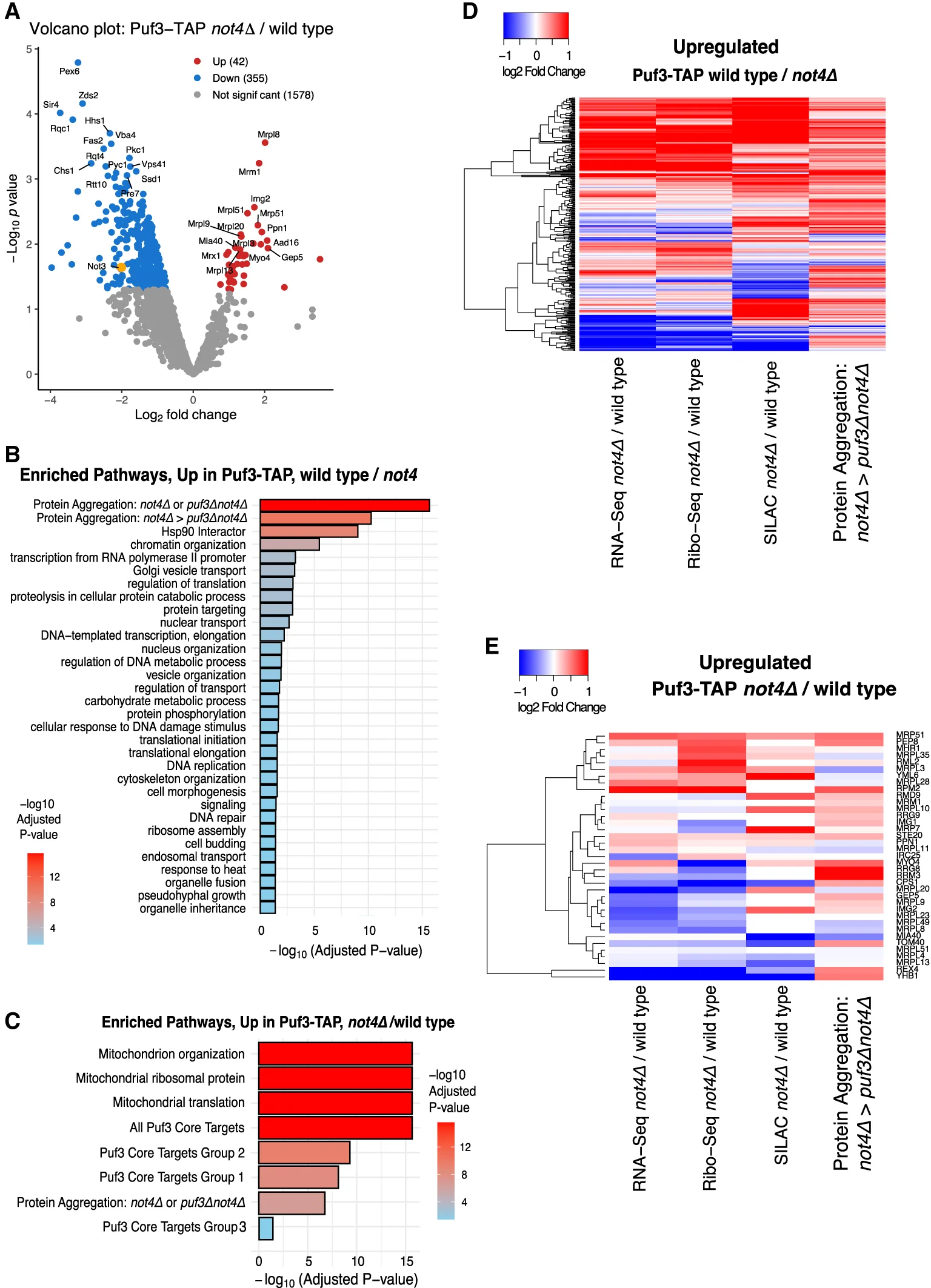
Characterization of the Puf3 interactome in *not4*Δ versus wild type. (*A*) Volcano plot representing the comparative Puf3-TAP purifications in wild type and *not4*Δ. (*B*) Enrichment analysis for the Puf3 interactome in wild type over *not4*Δ shows most significant enrichment of proteins that aggregate in *not4*Δ and Hsp90 interactors. (*C*) Enrichment analysis for the Puf3 interactome in *not4*Δ over wild type reveals enrichment of proteins associated with mitochondrial metabolism and Puf3 core targets. (*D*) Changes in expression extracted from previous data sets (Allen et al. 2021) corresponding to the proteins that are present in the Puf3 interactome from wild type cells but not mutant cells with generally no correlation. (*E*) Same as in *D* but corresponding to the proteins present in the interactome from mutant cells but not wild type cells.

We did the experiment with triplicate samples for a quantitative assessment of the Puf3 interactome in wild type and in *not4*Δ (Supplemental Fig. S5C). Principal component analysis indicated that one of the Puf3 purifications from wild type cells was very different than the other two (Supplemental Fig. S5D), hence we did not keep it for the further analyses. A volcano plot representing the comparative purifications in wild type and *not4*Δ is shown in Figure 5A, and the comparison of the wild type and *not4*Δ Puf3 interactomes is presented in Supplemental Table S5. Three hundred and fifty-five proteins were more enriched in the Puf3 interactome from wild type cells compared to that in mutant cells (Supplemental Table S5). On the other hand, 42 proteins were more enriched in the Puf3 interactome from *not4*Δ compared to wild type cells (Supplemental Table S5). Of the 355 proteins more significantly present in the Puf3 interactome from wild type cells compared to *not4*Δ cells, 292 of these were also more significantly present in the purification from wild type cells expressing Puf3-TAP compared to the untagged control (Supplemental Table S5). A striking finding was the presence of Not3 in the Puf3 interactome from wild type cells but not from *not4*Δ cells. As for the 42 proteins more significantly present in the Puf3 interactome from *not4*Δ cells compared to wild type cells, all 42 were also more significantly present in the purification from *not4*Δ cells expressing Puf3-TAP compared to the untagged control (Supplemental Table S5).

GO-term analysis of the proteins enriched in the Puf3 interactome from wild type cells compared to that of *not4*Δ revealed mainly proteins that tend to aggregate in the absence of Not4, Hsp90 interactors and proteins involved in chromatin organization (Fig. 5B; Supplemental Table S5). Instead, for the proteins enriched in the interactome from *not4*Δ cells, they were mostly mitochondrial proteins and proteins encoded by mRNA targets of Puf3 (Fig. 5C; Supplemental Table S5). This was interesting in light of the previous descriptions of both Puf3 and Not4 presence outside of mitochondrial membranes (Saint-Georges et al. 2008; Chen et al. 2023). Notably, Tom40, which is the central channel component of the TOM complex and functions as the entry gate for most mitochondrial proteins (for review, see Araiso et al. 2022) and is a Puf3 target, was in the interactome from mutant cells only.

In both the wild type and *not4*Δ Puf3 interactomes (Supplemental Table S5 for the wild type and Supplemental Table S5 for the *not4*Δ), known associated factors of Puf3, whether direct interactors or proteins whose interaction is mediated by mRNA, such as the poly(A) binding protein Pab1 (Klass et al. 2013) and the Hrr25 kinase (Bhondeley and Liu 2020), as well as mitochondrial proteins, consistent with the known importance of Puf3 for mitochondrial translation (Lee and Tu 2015; Lapointe et al. 2018; Wang et al. 2019; Hayashi et al. 2023), were significantly enriched. It is relevant to note that Not4 was identified as a protein significantly enriched in the Puf3 interactome from *not4*Δ cells compared to the untagged control, despite the absence of Not4 in this strain. Not4 was however in the mixed lysate from which the immunoprecipitation was performed. This reveals that interactions can indeed happen in the test tube, supporting our approach to focus only on proteins differentially purified from wild type or *not4*Δ cells. We compared the differential expression of proteins differentially present in the Puf3 interactomes from wild type and *not4*Δ using our previous SILAC, Ribo-seq and RNA-seq data in wild type and *not4*Δ (shown in Fig. 5D,E for the proteins for which we had the information from all three data sets [Allen et al. 2021]) and noted that there was no correlation between differential expression and differential co-purification from wild type or *not4*Δ.

Collectively, these findings indicate that the interactome of Puf3 is altered in *not4*Δ cells, and that this alteration is not a result of changes in protein abundance but is specifically linked to the loss of Not4 function.

## DISCUSSION

In this work we determine that Puf3 contributes to protein aggregation and temperature sensitivity in cells lacking Not4 (see model on Supplemental Fig. S5E). Not only does the deletion of Puf3 suppress partially temperature sensitivity and protein aggregation in yeast cells lacking Not4, but also Puf3 has a dominant negative impact on the growth of *not4*Δ cells. This negative impact of Puf3 in *not4*Δ correlates with an altered Puf3 interactome in *not4*Δ. Furthermore, we show that inappropriate translation elongation dynamics, in particular at rare arginine codons, associated with aberrant mRNA solubility, may be responsible for aggregation of new proteins due to aberrant Puf3 in *not4*Δ. In this study we looked at translation elongation dynamics (Ribo-seq) immediately upon Not4 depletion, with or without Puf3, but it should be noted that increased pausing at rare arginine codons is even more marked in cells lacking Not4 altogether (Allen et al. 2021). Although the mechanism by which Puf3 modulates this effect remains unclear, we determined that the deletion of Puf3 alone leads to reduced ribosome dwelling at rare arginine codons and, more broadly, at other nonoptimal codons. Hence, besides its role in *not4*Δ, our work also uncovers a new codon-specific impact of Puf3 deletion on translation elongation.

In our search for the mechanism linking Puf3 and Not4, we observed that Puf3 interactors specific to wild type cells (lost in *not4*Δ cells) were enriched for proteins that tend to aggregate in *not4*Δ. These findings suggest that Not4 is a “guardian” of Puf3's function that if left unattended can compromise protein homeostasis. Exactly how Not4 protects such a Puf3 regulatory axis is unclear. It could occur by Not4 controlling enzymes that posttranslationally modify Puf3 or by regulating components of the Puf3 interactome. However, it should be noted that the analysis of the aggregates with or without Puf3 was performed in a *not4*Δ mutant, which does not allow a dynamic analysis of Not4 ubiquitination of its targets and where indirect effects occurring in this mutant background cannot be excluded. In addition, Not4, like the other Not proteins, is present in condensates (Allen et al. 2021), as has also been proposed for Puf3 in glucose (Lee and Tu 2015), hence availability of proteins for interactions depending upon the status of the condensates is a possibility.

In this study, we were also able to determine that for specific mRNAs, changes in mRNA solubility in *not4*Δ upon the additional deletion of Puf3 coincided with changes in aggregation of encoded proteins. This was particularly interesting for a set of mRNAs for which protein homeostasis was improved when the mRNAs were less soluble (in the double mutant versus *not4*Δ), namely those mRNAs containing rare arginine codons. For these mRNAs, the action of Not4 and Puf3 are in opposition, consistent with opposing effects of Not4 and Puf3 on translation elongation at rare arginine codons. Hence, we believe that for these mRNAs, mRNA condensation resulting in decreased mRNA solubility might be regulated by ribosome dwelling at rare arginine codons and serve the purpose of appropriate protein folding or assembly during translation.

For specific mRNAs instead, loss of Puf3 opposes reduced mRNA solubility observed in *not4*Δ, and this coincides with decreased aggregation of the encoded proteins. We imagine that for these mRNAs, they become less soluble in *not4*Δ in the form of ribosome nascent chains (RNCs) with misfolded nascent chains, and Puf3 appears as a contributing factor to this nascent chain misfolding, maybe because of its relevance in elongation dynamics that we uncovered. It is notable that mRNAs in this category include chaperones such as Hsp90 (Hsc82 and Hsp82) (Supplemental Fig. S2C), suggesting that their production could be compromised in *not4*Δ and improved in the double mutant. Interestingly and consistent with this idea, the vast majority of the Hsp90 interactome shows significantly reduced protein aggregation in the double mutant relative to the single mutant when compared to all proteins (Supplemental Fig. S2C).

More available soluble chaperones and co-chaperones in the double *not4*Δ*puf3*Δ mutant relative to the single *not4*Δ mutant is likely to then indirectly prevent aggregation of many other proteins that are chaperone dependent, either co-translationally or posttranslationally. If it is co-translational, preventing aggregation of the nascent chain will also improve solubility of the encoding mRNA. If it is posttranslational, less protein aggregation will not correlate with changes in solubility of the encoding mRNAs. The translation termination factor eRF3 (encoded by *SUP35*) could be such a protein since its aggregation in *not4*Δ is reduced by the deletion of Puf3, but the solubility of its mRNA is not affected. In turn it could be that its function is compromised in *not4*Δ but less so in the double mutant. This may explain that we observe that the deletion of Puf3 in *not4*Δ reduces increased pausing at the stop codon in *not4*Δ.

Puf3 is known to interact with the Ccr4-Not complex and accelerate deadenylation during fermentation (Webster et al. 2019), but whether this known interaction might result in modulation of other functions of the Ccr4-Not complex has not been explored. In this report we observed that Not4 regulates the Puf3 interactome and Puf3's function, a regulation axis important to maintain protein homeostasis. We also determined that Puf3 from wild type cells but not from *not4*Δ cells, interacts with Not3, and that no other subunits of the Ccr4-Not complex were significantly enriched in the Puf3 interactome from wild type or *not4*Δ cells. The N-terminal domain of Not3 is homologous to that of Not5 which is the one that binds the ribosomal E site and modulates translation elongation dynamics. Maybe in *not4*Δ cells when Puf3 is present but no longer interacting with Not3, or in cells when just Puf3 is deleted, both conditions showing alterations in translation elongation dynamics, more Not3 is available to interact with the translating ribosome at the E site and contribute to ribosome dwelling. This is certainly an exciting avenue to investigate in the future.

Regardless of the exact mechanisms linking Puf3 and the Not proteins, this work clearly supports the idea that Puf3 has a broad role in regulation of translation elongation dynamics that impacts all mRNAs and not only its targets, and that is connected to the functions of the Ccr4-not complex, especially of Not4. Moreover, the findings of this work indicating that Puf3 contributes to some, but not all protein aggregation in *not4*Δ, leaves open the door to identifying other Ccr4-Not interacting RBPs, that together with Puf3 might mediate the global loss of protein homeostasis in *not4*Δ.

## MATERIALS AND METHODS

### Yeast strains and growth conditions

Strains used in this manuscript were either from Euroscarf and derived from BY4739 (*MAT*α *his3 leu2 ura3 lys2*), wild type (MY3672) and *not4*Δ (MY3417, isogenic except *not4::KANMX4*) used for ribosome profiling previously (Allen et al. 2021), *puf3*Δ (MY8058, isogenic except *puf3::KANMX4*) and *not4*Δ*puf3*Δ generated by PCR from MY8058 (leading to MY12455, isogenic to MY3672 except *puf3:KANMX4 not4::NATMX4*). The Not1 (MY13517) and Not4 (MY13771) degron strains have been described previously (Allen et al. 2023). *puf3*Δ was created by PCR in the Not1 and Not4 degron strains (leading to MY14441 and MY14401) and as well as in the isogenic wild type TIR-expressing strain (MY13472, leading to MY14416), by PCR. All strains were verified by PCR. The strain expressing Puf3-Tap-tag (indicated Puf3-TAP) was from Euroscarf (SC1249, here called MY8057), and the *NOT4* gene was deleted in this strain by PCR (leading to MY12356). All strains were verified by PCR and are listed in Supplemental Table S1.

All experiments were performed with cells growing in YPD or in selective SC medium for experiments with cells transformed with plasmids.

Not1 and Not4 depletions were obtained by addition of auxin (3-indoleacetic acid, stock solution at 250 mM in EtOH 100%) at 1 mM final for 15 min to exponentially growing cells diluted after an overnight culture in glucose rich medium (YPD) when they reached an OD_600_ of 0.8–1.

For the spot growth tests, cells were collected from plates and resuspended in appropriate media and then adjusted to an OD_600_/mL of 0.1–0.2 from which four- or fivefold serial dilutions were prepared (40 in 160 μL) and 4 or 5 μL of each dilution were spotted on appropriate plates with or without cycloheximide (6 μg/mL). For all phenotypic experiments, several independent isolates were analyzed, and the results for isolates that are not in the main figures can be found in the Supplemental Material and in the raw data files.

### Plasmids and oligos

Plasmids and oligonucleotides used in this study are listed in Supplemental Table S1.

For the plasmid expressing the tRNA having the ACG anticodon that is converted to ICG in the mature tRNA, a PCR amplification using genomic DNA from a wild type strain (MY3672) was performed using primers 1419 and 1420. The generated fragment was digested with *Eco*RI and *Not*I together with the pRS426 plasmid. Both insert and vector were ligated using the T4 DNA ligase (NEB) and transformed in *Escherichia coli* competent cells. Transformants were selected on LB ampicillin plates and clones were tested by digestion and confirmed by sequencing. This created plasmid pMAC1452. This plasmid was then digested with *Nco*I and *Not*I together with the pRS416 vector and further cloning steps were proceeded as described above to create pMAC1467.

For the plasmid expressing the tRNA having the anticodon UCG, the tRNA gene was amplified from DNA of a wild type strain (MY3672) using the primers 1419 and 1428 and 1427 and 1420 to mutate the CGT to CGA. The generated fragment was further amplified using 1419 and 1420 primers and the amplicon was digested with *Eco*RI and *Not*I. The pRS424 vector was digested using *Eco*RI and *Not*I. Both fragment and insert were ligated using the T4 DNA ligase, and we proceeded with further cloning steps as described above to create pMAC1457. This plasmid was then digested with *Nco*I and *Not*I together with pRS416 and again we proceeded with further cloning steps as described above to create pMAC1466.

To create pMAC1489 expressing Puf3 with a C-terminal flag-tag from its own promoter with its own terminator, *PUF3* DNA was amplified from wild type (MY3672) genomic DNA by PCR using primers 1488 and 1489 for the 5′ fragment and 1490 and 1491 for the 3′ fragment. The vector pRS416 was digested with *Eco*RI and *Not*1, and the two fragments with the digested vector were assembled using a Gibson Assembly Cloning Kit (NEB EE5510S). The plasmid was verified by digestion and sequencing.

### Postalkaline lysis for analysis of total protein levels

Exponentially growing cells expressing Puf3-TAP were pelleted and were resuspended in 0.1 M NaOH and incubated for 10 min at room temperature (RT). After a quick spin in a microfuge, the cell pellet was resuspended in 2× sample buffer (SB) followed by standard SDS-PAGE and western blotting with antibodies to the Protein A (Sigma P1291) and Gapdh (Thermo Fisher Scientific MA5-15738) for loading control.

### Test of protein integrity

Exponentially growing yeast cells expressing Puf3-TAP were harvested at 1 OD_600_/mL (50 mL of cells) by spinning for 15 min at 3500 rpm at RT and kept at −20°C. Before breaking, pelleted cells were resuspended with 400 μL of lysis buffer (20 mM Hepes pH 7.4, 100 mM KOAc, 1× Ribonucleoside Vanadyl Complex [RVC, ribonuclease inhibitor, NEB S1402S], 1× protease inhibitor [Pierce Protease Inhibitor Tablets, EDTA-free, Thermo Fisher Scientific A32965]). Cells were broken using glass beads by vortexing for 10 min at 4°C. To pellet glass beads and cellular debris, samples were spun for 20 min at 13,000 rpm at 4°C, then clear lysates were transferred into new tubes. Protein concentration was determined by a Bradford assay, and all samples were adjusted to the same concentration. One hundred microliters of adjusted samples were mixed with 50 μL 4× SB. Serial dilutions starting with 10 μL of lysate containing equal amounts of total protein were separated by SDS-PAGE and analyzed by western blot using antibodies to Protein A (Sigma P1291) and Gapdh (Thermo Fisher Scientific MA5-15738) for loading control.

### Preparation of total and soluble RNA

Total RNA was prepared either by the hot acid phenol method (Collart and Oliviero 2001) or cells were prepared and lysed as for polysome fractionation (Panasenko and Collart 2012) and total RNA was prepared from the lysate. Briefly, for total RNA, cell pellets from 30 mL of exponentially growing yeast were resuspended with 400 μL of TES buffer (10 mM Tris HCl pH 7.5, 10 mM EDTA and 0.5% SDS) to which 400 μL of acid phenol were added. After vortexing and incubation for 10 min at 65°C, total RNA was extracted. For soluble RNA, cell pellets from 30 mL of exponentially growing yeast were resuspended in 400 μL of lysis buffer (20 mM Hepes, 20 mM KCl, 10 mM MgCl_2_ 1% Triton X-100, 1 mM PMSF, 1 mM DTT, 1× protease inhibitor [Pierce Protease Inhibitor Tablets, EDTA-free, Thermo Fisher Scientific A32965]) to which 200 μL of glass beads were added. Cells were vortexed at 4°C for 15 min and spun at 15,000*g* for 1 min. The supernatant was transferred to a new tube and spun 20 min at 4°C at 15,000*g*. The supernatant was combined with 400 μL of acid phenol for further RNA extraction as for the total RNA pool.

Wild type (MY3672) and *not4*Δ (MY3417) total and soluble RNAs were prepared and analyzed by RNA-seq previously, and soluble RNAs from wild type (MY3672) and *not4*Δ (MY3417) were also analyzed previously by Ribo-seq (Allen et al. 2021, 2023). In this study, soluble RNAs from wild type (MY3672), *puf3*Δ (MY8058) and *not4*Δ*puf3*Δ (MY12455) were prepared and analyzed similarly by RNA-seq.

### Soluble and total RNA-seq

The total and soluble RNA was prepared from 50 mL of exponentially growing cells in YPD. Libraries were generated as reported (Allen et al. 2023). In brief, 7.5 μg RNA were subject to random fragmentation (5 min at 80°C in fragmentation buffer: 40 mM Tris Acetate pH 8.1, 100 mM KOAc and 30 mM MgOAc) prior to making the HT-Seq libraries. Samples were purified using 1.8× volumes of RNACleanXP beads (Beckman Coulter) following re-phosphorylation of 5′OH sites at 37°C for 60 min with 5 Units of T4 Polynucleotide kinase (PNK, NEB). Subsequently, the reaction was purified using Phenol:Chloroform:Isoamyl Alcohol (24:25:1), followed by sodium acetate-ethanol precipitation.

For HT-Seq libraries: 7.5 μg RNA was ligated overnight at 16°C to r5P_RNA_MPX oligo (CrArCrGrArCrGrCrUrCrUrUrCrCrGrArUrCrU rXrXrXrXrXrX rNrNrNrNrNrNrNrN) carrying a sample barcode (rX) and unique molecular identifiers (rN). Ligase was deactivated using 5 mM EDTA and heated at 65°C for 10 min (up to X individual barcoded RNA ligations were pooled) and subsequently purified using 1.8 X volumes of RNAClean XP beads (Beckman Coulter). Ligated RNA was then reverse transcribed using random hexamer (5Pseq-RT, GTGACTGGAGTTCAGACGTGTGCTCTTCCGATCTNNNNNN, 20 μM) and oligo-dT (5Pseq-dT, GTGACTGGAGTTCAGACGTGTGCTCTTCCGATCTTTTTTTTTTT at 0.05 μM) oligos to prime. After, the remaining RNA was degraded using NaOH. Ribosomal RNA was removed using previously described rRNA DNA oligo depletion mixes, following a duplex-specific nuclease (DSN, Evrogen) digestion. rRNA depleted cDNA was amplified by PCR (17 cycles) and the final product was enriched for fragments with the range of 300–500 nt using Ampure XP. Size selected HT libraries were quantified by fluorescence (Qubit, Thermo Fisher Scientific), and the size was estimated using an Agilent Bioanalyzer and sequenced using a NextSeq500 Illumina Sequencer (75 cycles High Output Kit).

Sequencing files were demultiplexed using bcl2fastq v2.20.0.422 (one mismatch, minimum length 35 nt), and adapters were trimmed using cutadapt 2.3 at default settings, allowing one mismatch and a minimum read length of 35 nt. In addition to standard illumine dual index (i5, i7), the inline sample and UMI barcode was analyzed using Umitools. Reads were mapped to the concatenated genome of *Saccharomyces cerevisiae* (R64-1–1) using STAR.

Second read enables to split reads between oligo-dT or random primer. That information was not used in the current analysis. CDS positions were defined with the Ensembl gff version 94 for *S. cerevisiae* (R64-1–1). Counts in *S. cerevisiae* were calculated by aggregating RNA-seq read counts overlapping CDS positions.

### Solubility

We define this as the log fold change produced by DESeq2, dividing RNA-seq counts for the soluble RNA fraction in a given sample by the corresponding counts for the total RNA fraction of the same sample. The solubility of deletion and depletion strains is then corrected to wild type basal solubility.

### Ribosome profiling (Ribo-seq)

Soluble RNAs from wild type (MY13472), *puf3*Δ (MY14373), Not1 degron (MY13517), Not4 degron (MY13771), Not1 degron with *puf3*Δ (MY14441) and Not4 degron with *puf3*Δ (MY 14401) were analyzed by Ribo-seq in biological triplicates with some modifications compared to the previous Ribo-seq analysis (Panasenko et al. 2019), at the BioCode: RNA to Proteins Core Facility of the Faculty of Medicine (University of Geneva).

For cell preparation, exponentially growing cells were collected at an OD_600_/mL of 0.8–1, and before auxin treatment, an aliquot of cells was taken to verify the protein levels prior to depletion. Auxin was added at a final concentration of 1 mM for 15 min at 30°C under agitation, and an aliquot of cells was taken to check for efficient protein depletion. Before cell collection by centrifugation, cycloheximide was added in the media at 0.1 mg/mL final concentration and cells were spun for 3 min at 4600*g* at 4°C. Pelleted cells were resuspended in 30 mL of polysome buffer (PB: 20 mM Tris, pH 7.4, 150 mM NaCl, 5 mM MgCl_2_, 1 mM DTT, 0.1 mg/mL cycloheximide). Cells were spun for 3 min at 4600*g* at 4°C. The cell pellet was resuspended in fresh PB and transferred to a new tube prior to spinning. The pellets were kept frozen at −80°C.

The degron protein levels were checked by postalkaline lysis, standard SDS-PAGE and western blotting with antibodies to myc, from cells collected before and after auxin addition. Postalkaline lysis involves incubating yeast cells in 0.1 M NaOH for 5 min at RT, pelleting, and suspending the pellet in 2× SB. After confirmation of protein depletion, the frozen cell pellet was processed further for Ribo-seq and soluble RNA extraction from extracts prepared as described previously (Panasenko et al. 2019).

Soluble RNAs were isolated from the same extracts that were used to obtain RPFs. Three volumes of QIAzol (Qiagen 79306) were added to 80 μL of extracts, mixed and proceeded to RNA purification with Direct-Zol RNA Mini Prep Plus kit (Zymo Research R2070).

RNAs were sent to the iGE3 Genomic Platform (University of Geneva) for stranded mRNA library preparation and sequencing. Quality control of RNAs was checked by Bioanalyzer. RNAs with quality higher than RIN: 9.80 were used for library preparation. Libraries were sequenced on an Illumina NovaSeq 6000, single-reads, 1 × 100 bp.

To obtain ribosome footprints, 100 μL of total extracts containing 500 μg of total RNA were treated with RNase I (Ambion, 100 units/μL AM2294) with a concentration of 1600 U per 1 mg of total RNA, for 1 h at 20°C with slow agitation. Ten microliters of SUPERase•In RNase Inhibitor (Ambion AM2694) was added to stop nuclease digestion. Monosomes were isolated by size-exclusion chromatography using MicroSpin S-400 HR columns (Cytiva 27514001). Three volumes of QIAzol (Qiagen 79306) were added to the S-400 eluate, mixed and proceeded to RNA purification with the Direct-Zol RNA Mini Prep Plus kit (Zymo Research R2070).

For Ribo-seq library preparation, RPFs (25–34 nt) were size-selected by electrophoresis using 15% TBE-Urea polyacrylamide gel electrophoresis (PAGE) and two RNA markers, 25-mer (5′ AUGUACACGGAGUCGAGCACCCGCA 3′) and 34-mer (5′ AUGUACACGGAGUCGAGCACCCG CAACGCGAAUG 3′). After dephosphorylation with the T4 Polynucleotide Kinase (NEB M0201S), the adapter Linker-1 (5′ rAppCTGTAGGCACCATCAAT/3ddC/3′) was ligated to the 3′ end of the RPF using T4 RNA Ligase 2 (NEB M0242S). The ligated products were purified using 10% TBE-Urea PAGE. Ribosomal RNAs were subtracted using RiboCop rRNA Depletion Kit V2 Yeast (Lexogen 190). The adapter Linker-1 was used for priming reverse transcription with the primer Ni-Ni-9 (5′AGATCGGAAGAGCGTCGTGTAGGGAAAGAGTGTAGATCTCGGTGGTCGC5CACTCA5TTC AGACGTGTGCTCTTCCGATCTATTGATGGTGCCTACAG 3′) containing two internal 18-atom hexa-ethylene glycol (HEG) chains, using ProtoScript II Reverse Transcriptase (NEB M0368S). Reverse transcribed products were purified using 10% TBE-Urea PAGE. The cDNA was circularized with CircLigase II ssDNA Ligase (Epicentre CL9021K). The final libraries were generated by PCR using forward index primers. Amplified libraries were purified using 8% TBE-PAGE and analyzed with TapeStation High Sensitivity. Libraries were sequenced on an Illumina NovaSeq 6000, single-reads, 1 × 100 bp.

For *not4*Δ and associated wild type Ribo-seq samples, data were analyzed as previously described (Allen et al. 2021).

Published Ribo-seq data for *puf3*Δ were downloaded to be re-analyzed from Wang et al. (2019), and new Ribo-seq data were generated for wild type, Not1 depletion (*Not1d*), Not4 depletion (*Not4d*), *puf3*Δ, *Not1d puf3*Δ and *Not4d puf3*Δ; fastq files were adaptor stripped using cutadapt (Martin 2011). Only trimmed reads were retained, with a minimum length of 15 and a quality cutoff of 2 (parameters: -a CTGTAGGCACCAT ‐‐trimmed-only ‐‐minimum-length = 15 ‐‐quality-cutoff = 2). Histograms were produced of ribosome footprint (RPF) lengths that were very homogeneous with highest reads between 26 and 29 that were kept for the analysis. Reads were mapped using default parameters, with HISAT2 (Kim et al. 2015) to R64-1-1, using Ensembl release 84 gtf for transcript definitions. UTR definitions were taken from the *S. cerevisiae* Genome Database and a standard region of 100 bp was used where a gene's UTR was not defined. A full set of 6692 CDSs were established for R64-1-1 Ensembl release 84 and extended by the same UTR sequences defined above. Definitions of rRNA loci were taken from UCSC RepeatMasker tracks for R64-1-1, and all reads mapping to these regions were removed. The filtered reads were then mapped to this transcriptome with Bowtie2 (Langmead and Salzberg 2012) using default parameters. Only unique alignments to transcripts were retained. For all downstream analyses, dubious ORFs were filtered to leave 5929 transcripts. The A/P site position of each read was predicted by riboWaltz (Lauria et al. 2018) and aggregated over all transcripts. Differential expression was performed using DESeq2 (Love et al. 2014).

Where RDOs are reported for E, P, A sites, a count on a given codon is taken to be the number of E, P, A sites covering it, respectively. These values are divided by the mean codon coverage for every respective CDS included in the analysis and averaged for each codon type. Transcripts used in the analysis of RDOs were restricted to those with RPKMs >1 across all samples, leaving 4060 transcripts. The log_2_ ratio of deletions or depletions for RDOs were taken relative to wild type for each codon. When reporting a pairwise correlation, RDO values for each codon were calculated for each sample and correlated across all 61 codons. Optimal codons were defined as the 15 codons with the highest tRNA adaptation index (tAI) and nonoptimal codons as the 15 with the lowest tAI (Pechmann and Frydman 2013).

### Quantification and statistical analysis

Reported correlations were the Pearson's product-moment correlation coefficient and were used as test statistics to generate the associated *P*-value by *t*-test. All correlations and correlation tests were performed on groups of at least 30 in size. Enrichment of gene sets is defined via FDR after Benjamini–Hochberg adjustment from *P*-values generated using a hypergeometric test. All *t*-tests were performed on sample sizes of 30 or higher, where the Central Limit Theorem applies regarding the normality assumption. We used Welch's *t*-test in all cases, rather than the Student's *t*-test, resulting in a more conservative *P*-value, which is more reliable where variances and sample sizes are unequal.

### tRNA microarrays

To determine the fraction of charged tRNAs, we followed the procedure described in Polte et al. (2019). Briefly, total RNA was isolated in mild acidic conditions (pH 4.5) which preserves the aminoacyl-group. Each sample was split into two aliquots and one was oxidized with periodate to which the charged tRNAs remain intact. Following subsequent deacylation (100 mM Tris, pH 9.0 at 37°C for 45 min), it was hybridized to a Cy3-labeled RNA/DNA stem–loop oligonucleotide. The second aliquot was deacylated to obtain the total tRNA and hybridized to Atto647-labeled RNA/DNA stem–loop oligonucleotide. Both labeled aliquots were mixed in equimolar amounts, spiked-in with standards and analyzed on the same tRNA microarrays. The slides for the microarrays contained tRNA probes covering the full-length sequence of cytoplasmic tRNA species, as described previously (Allen et al. 2021).

For tRNA abundance, the total RNA was isolated at alkaline pH to simultaneously deacylate all tRNAs. tRNAs labeled with Cy3-labeled RNA/DNA stem–loop oligonucleotide were hybridized on the same microarray with tRNAs isolated from a wild type strain labeled with Att647-labeled RNA/DNA stem–loop oligonucleotide. The arrays were normalized to spike-in standards and processed and quantified with in-house python scripts, as described previously (Polte et al. 2019).

### Preparation of soluble extracts and aggregated proteins

Protein aggregates were isolated as described previously (Koplin et al. 2010; Panasenko and Collart 2012). 50 OD_600_ of cells growing exponentially were collected by centrifugation at RT (4000 rpm, 5 min). Cell pellets were washed with H_2_O and transferred to Eppendorf tubes. The pellets were then resuspended in 0.5 mL of lysis buffer (20 mM NaPi pH 6.8, 1 mM EDTA, 0.1% Tween 20, 10 mM DTT, 1 mM PMSF, 1× protease inhibitor [Pierce Protease Inhibitor Tablets, EDTA-free, Thermo Fisher Scientific A32965], 3 mg/mL Zymolase T20) at 30°C for 20 min. Samples were put on ice and sonicated (10 counts, three times, 40% intensity). Samples were spun for 20 min at 200*g* at 4°C. The supernatant was kept, and a Bradford assay was done to determine protein concentration. All samples were adjusted to the same protein concentration. An aliquot of the total extract was kept for total extract analysis (mixed with 2× SB) and the remainder of the samples was spun for 20 min at 16,000*g* at 4°C. Pellets were washed in 0.5 mL of WB-NP (20 mM NaPi pH 6.8, 1 mM PMSF, 1× protease inhibitor [Pierce Protease Inhibitor Tablets, EDTA-free, Thermo Fisher Scientific A32965] and 2% of NP-40) and sonicated (10 counts, one time 40% of intensity). This was repeated once and pellets were resuspended in 0.5 mL of WB (20 mM NaPi pH 6.8, 1 mM PMSF [Pierce Protease Inhibitor Tablets, EDTA-free, Thermo Fisher Scientific A32965]) and sonicated (10 counts, one time, 40% intensity). Samples were spun for 20 min at 16,000*g* at 4°C and pellets were resuspended in 2× SB. Samples were boiled at 100°C for 5 min and loaded on gradient SDS gels (4%–12% NuPAGE bis-acrylamide, Thermo Fisher Scientific) for silver nitrate analysis. All analyses by silver staining were performed in at least three biological triplicates, and all gels besides the one provided in the main figure are provided in the raw data files. Quantification of the gels was performed by ImageJ2 version 2.16.0/1.54p. Band intensities were normalized to wild type controls. Fold changes relative to wild type were log_2_-transformed to stabilize variance and approximate normality. Replicate four in the *not4*Δ*puf3*Δ condition appeared to be a significant outlier, with a log_2_ fold change value of −0.74, whereas the other three replicates were densely clustered ∼0.497 with a standard deviation of 0.076, placing the value in replicate four more than 16 SDs from the mean of the other three. This was further confirmed by Dixon's *Q* test for outliers (*Q* = 0.89, *P* = 0.019), and the value was therefore excluded from downstream analyses. Statistical analysis was performed using one-sided, one-sample Welch's *t*-test against zero (corresponding to wild type) to assess significant deviations from wild type in each mutant. One-sided, paired, two-sample Welch's *t*-tests were used for all pairwise comparisons between mutants. *P*-values were annotated using standard significance symbols (*P < 0.05, **P < 0.01, ***P < 0.001). Boxplots in Figure 2A display the distribution of log_2_ fold changes, with individual replicates overlaid, pairwise significance indicated above the boxes, and comparisons against wild type shown below.

### Mass spectrometry characterization of aggregated proteins

The quantitative proteomic analysis was performed by the LC-MS at the Proteomics Core Facility of the Faculty of Medicine (University of Geneva).

Samples of the protein aggregates were prepared as described above, except that an extra washing in WB was done to eliminate traces of detergent, and the aggregate samples were not resuspended in 2× SB but kept as dried pellets at −20°C.

Cell pellets were resuspended in 50 μL of 0.1% RapiGest Surfactant (Waters) in 50 mM ammonium bicarbonate (AB). Samples were heated for 5 min at 100°C and sonicated (6 × 30 sec) at 70% amplitude and 0.5 pulse. Samples were kept 30 sec on ice between each cycle of sonication. Samples were centrifuged for 10 min at 14,000*g*. Sample volume was adjusted to 100 μL with 0.1% RapiGest in 50 mM AB. Two microliters of dithioerythritol (DTE) 50 mM were added and the reduction was carried out at 37°C for 1 h. Alkylation was performed by adding 2 μL of iodoacetamide 400 mM during 1 h at RT in the dark. Overnight digestion was performed at 37°C with 8 μL of freshly prepared trypsin (Promega: 0.1 μg/μL in 50 mM AB). To remove RapiGest, samples were acidified with TFA, heated at 37°C for 45 min and centrifuged for 10 min at 17,000*g*. Supernatants were then desalted with a C18 microspin column (Harvard Apparatus) according to manufacturer's instructions, completely dried under speed-vacuum and stored at −20°C.

#### ESI-LC-MSMS

Samples were dissolved in 19 μL of loading buffer (5% CH_3_CN, 0.1% FA). Biognosys iRT peptides were added to each sample and 2 μL of peptides were injected on the column. LC-ESI-MS/MS was performed on an Orbitrap Fusion Lumos Tribrid Mass Spectrometer (Thermo Fisher Scientific) equipped with an Easy nLC1200 liquid chromatography system (Thermo Fisher Scientific). Peptides were trapped on an Acclaim PepMap 100, C18, 3 μm, 75 μm × 20 mm Nano-Trap Column (Thermo Fisher Scientific) and separated on a 75 μm × 500 mm, C18 ReproSil-Pur (Dr. Maisch GmBH), 1.9 μm, 100 Å, homemade column. The analytical separation was run for 135 min using a gradient of H__2__O/FA 99.9%/0.1% (solvent A) and CH__3__CN/H__2__O/FA 80.0%/19.9%/0.1% (solvent B). The gradient was run from 8% B to 28% B in 110 min, then to 42% B in 25 min, then to 95% B in 5 min with a final stay of 20 min at 95% B. Flow rate was 250 nL/min and total run time was 160 min. Data-independent acquisition (DIA) was performed with MS1 full scan at a resolution of 60,000 (FWHM), followed by 30 DIA MS2 scan with sequential fix isolation windows of 28 m/z. MS1 was performed in the Orbitrap with an AGC target of 1 × 10^6^, a maximum injection time of 50 msec and a scan range from 400 to 1240 m/z. DIA MS2 was performed in the Orbitrap using higher-energy collisional dissociation (HCD) at 30%, an AGC target of 1 × 10^6^ and a maximum injection time of 54 msec.

#### Data analysis

DIA raw files were loaded into Spectronaut v.18 (Biognosys) and analyzed by directDIA using default settings. Briefly, data were searched against the *S. cerevisiae* database (UniProt release 2023-08, 6060 entries). Trypsin was selected as the enzyme, with one potential missed cleavage. Variable amino acid modifications were oxidized methionine. Fixed amino acid modification was carbamidomethyl cysteine. Both peptide precursor and protein FDR were controlled at 1% (*Q* < 0.01). Single-hit proteins were excluded. For quantitation, the Protein LFQ method was set to “QUANT 2.0,” “only protein group specific” was selected as proteotypicity filter and normalization was set to “none.” The quantitative analysis was performed with Spectronaut; proteins were considered to have significantly changed in abundance with a *Q* ≤ 0.05 and an absolute fold change FC ≥ |1.5| (log_2_FC ≥ |0.58|).

For Figure 4C, to assess the relative contributions of length and codon content on aggregation, a linear model was formulated. For each codon “c,” its frequency in each transcript with a protein product present in aggregation data for *not4*Δ and *not4*Δ*puf3*Δ was calculated and then normalized to the transcript length in amino acids, giving the proportion of codon “c.” Separately for each codon “c,” a linear model was then constructed across transcripts with the relative aggregation rate log_2_ *not4*Δ/*not4*Δ*puf3*Δ of their protein products as the dependent variable, and the proportion of codon “c” in each transcript and the natural logarithm of the length of each transcript as dependent variables. The contribution of the proportion of each codon “c” across transcripts to the change in aggregation rate between *not4*Δ and *not4*Δ*puf3*Δ in their protein products, while controlling for transcript length, was then calculated as the coefficient of the codon proportion variable in the linear model for codon “c.” Assessing this value across all codons quantifies their relative contribution to change in aggregation between strains, while controlling for transcript length.

### Puf3 purification and mass spectrometry

Exponentially growing yeast cells were harvested at 1 OD_600_/mL (1.8 L of cells) for 15 min at 3500 rpm at RT and kept at −20°C. Before breaking, the cells were pelleted and resuspended with 5 mL of lysis buffer (20 mM Hepes pH 7.4, 100 mM KOAc, 1× Ribonucleoside Vanadyl Complex [RVC, ribonuclease inhibitor, NEB S1402S], 1× protease inhibitor [Pierce Protease Inhibitor Tablets, EDTA-free, Thermo Fisher Scientific A32965]) and mixed appropriately. Cell mixtures were then broken using glass beads by vortexing for 10 min at 4°C. To pellet glass beads and cellular debris, samples were centrifugated for 20 min at 13,000 rpm, 4°C and clear lysates were transferred into new tubes.

IgG coupled superparamagnetic beads were prepared accordingly to Namane and Saveanu (2022).

Twenty microliters of IgG coupled beads per sample were washed four times in the extraction buffer (20 mM Hepes pH 7.4, 100 mM KOAc, 0.5% Triton X-100). Beads were resuspended in 100 μL of extraction buffer (per sample) and added to the clear lysates. Samples were put on rotation at 4°C for 2 h. After binding, beads were washed five times in the extraction buffer. One extra washing was performed in the extraction buffer without Triton X-100. Elution was performed by adding 50 μL of SDS elution buffer (10 mM Tris-HCl, 1 mM EDTA, 2% SDS) and incubating for 10 min at 65°C.

Five microliters of the eluate was taken to perform a western blot with an antibody against the Protein A (Sigma P1291). The remainder of the sample was precipitated with methanol-chloroform according to Namane and Saveanu (2022). Dry pellets were conserved at −20°C before proceeding to mass spectrometry.

Samples were prepared using iST kits (PreOmics) according to the manufacturer's instruction. Briefly, pellets were re-suspended in 50 μL of provided lysis buffer and proteins were denatured, reduced and alkylated during 10 min at 60°C. The resulting slurries (beads and lysis buffer) were transferred to dedicated cartridges and proteins were digested with a Trypsin/LysC mix for 2 h at 37°C. After two cartridge washes, peptides were eluted with 2 × 100 μL of provided elution buffer. Samples were finally completely dried under speed vacuum and stored at −20°C.

#### LC-ESI-MSMS

Samples were dissolved in 20 μL of loading buffer (5% CH_3_CN, 0.1% FA) and 4 μL were injected on a column. LC-ESI-MSMS was performed on a Q-Exactive HF Hybrid Quadrupole-Orbitrap Mass Spectrometer (Thermo Fisher Scientific) equipped with an EASY-nLC 1000 liquid chromatography system (Thermo Fisher Scientific). Peptides were trapped on an Acclaim PepMap 100 C18, 3 μm, 75 μm × 20 mm Nano-Trap Column (Thermo Fisher Scientific) and separated on a 75 μm × 250 mm, C18, 2 μm, 100 Å Easy-Spray column (Thermo Fisher Scientific). The analytical separation was run for 90 min using a gradient of H_2_O/FA 99.9%/0.1% (solvent A) and CH_3_CN/FA 99.9%/0.1% (solvent B). The gradient was run as follows: 0–5 min 95% A and 5% B, then to 65% A and 35% B for 60 min, and 5% A and 95% B for 20 min at a flow rate of 250 nL/min. Full scan resolution was set to 60,000 at m/z 200 with an AGC target of 3 × 10^6^ and a maximum injection time of 60 msec. Mass range was set to 400–2000 m/z. For data dependent analysis, up to 20 precursor ions were isolated and fragmented by higher-energy collisional dissociation HCD at 27% NCE. Resolution for MS2 scans was set to 15,000 at m/z 200 with an AGC target of 1 × 10^5^ and a maximum injection time of 60 msec. Isolation width was set at 1.6 m/z. Full MS scans were acquired in profile mode, whereas MS2 scans were acquired in centroid mode. Dynamic exclusion was set to 20 sec.

Database search peak lists (MGF file format) were generated from raw data using the msConvert conversion tool from ProteoWizard. The peaklist files were searched against the *S. cerevisiae* reference proteome database (UniProt 2025-03, 6066 entries), the Puf3-TAP bait protein sequence (*S.* Genome Database [SGD], https://www.yeastgenome.org/) and an in-house database of common contaminant using Mascot (Matrix Science; version 2.6.2). Trypsin was selected as the enzyme, with one potential missed cleavage. Precursor ion tolerance was set to 10 ppm and fragment ion tolerance to 0.02 Da. Variable amino acid modifications were oxidized methionine, deaminated (NQ) phosphorylated (STY) and GG (K). Fixed amino acid modification was carbamidomethyl cysteine. The Mascot search was validated using Scaffold 5.3.3 (Proteome Software). Peptide identifications were accepted if they could be established at >33.0% probability to achieve an FDR <0.1% by the Percolator posterior error probability calculation (Kall et al. 2008). Protein identifications were accepted if they could be established at >99.0% probability to achieve an FDR <1.0% and contained at least two identified peptides. Protein probabilities were assigned by the Protein Prophet algorithm (Nesvizhskii et al. 2003). Proteins that contained similar peptides and could not be differentiated based on MS/MS analysis alone were grouped to satisfy the principles of parsimony.

For the principal component analysis (PCA) plots and differential expression analysis, normalized abundance values of mass spectrometry data from Puf3–TAP affinity purifications were log_2_-transformed after addition of a pseudocount of one to stabilize variance and normalize signal distributions across samples. PCA was then performed using the prcomp function in R and the first two principal components were plotted including all samples.

To carry out differential expression analysis, samples were categorized into three experimental groups: No-Tag controls (*n* = 3), Puf3–TAP in wild type (*n* = 2), and Puf3–TAP in *not4*Δ (*n* = 3). A linear model was fitted using limma (Ritchie et al. 2015) (with a design matrix specifying these groups). Contrasts were defined to assess differential protein abundance between Puf3–TAP and No-Tag control, and between Puf3–TAP *not4*Δ and Puf3–TAP. Moderated *t*-statistics were computed using the empirical Bayes framework implemented in limma, providing robust variance estimation across proteins.

*P*-values were derived from these *t*-statistics and proteins were selected as upregulated if they showed a fold change >1 and a *P* < 0.05 in both comparisons, *not4*Δ/wild type (wild type) and *not4*Δ/NoTag. Proteins were selected as downregulated if they showed a fold change >1 and a *P* < 0.05 in both comparisons, wild type/*not4*Δ and wild type/NoTag.

The upregulated set and the downregulated set were then subjected to an enrichment test against gene ontology gene sets, with a handful of custom gene sets derived from our own data. This was done via a hypergeometric test, using all annotated proteins.

## DATA DEPOSITION

The new Ribo-seq data are accessible under GSE303644, the mass spectrometry data are in the Pride partner repository under PXD069853, and the tRNA microarray data are accessible under accession number GSE305765 in the Gene Expression Omnibus (GEO) database. The *not4*Δ RNA-seq and degron RNA-seq are already online at https://www.ncbi.nlm.nih.gov/geo/query/acc.cgi?acc=GSE168290 and https://www.ncbi.nlm.nih.gov/geo/query/acc.cgi?acc=GSE193912. All code used in the manuscript is available at https://github.com/georgeallenunige/NotTranslation.

## SUPPLEMENTAL MATERIAL

Supplemental material is available for this article.
